# *In silico* Prediction, Characterization, Molecular Docking, and Dynamic Studies on Fungal SDRs as Novel Targets for Searching Potential Fungicides Against Fusarium Wilt in Tomato

**DOI:** 10.3389/fphar.2018.01038

**Published:** 2018-10-22

**Authors:** Mohd Aamir, Vinay Kumar Singh, Manish Kumar Dubey, Mukesh Meena, Sarvesh Pratap Kashyap, Sudheer Kumar Katari, Ram Sanmukh Upadhyay, Amineni Umamaheswari, Surendra Singh

**Affiliations:** ^1^Laboratory of Mycopathology and Microbial Technology, Centre of Advanced Study in Botany, Institute of Science, Banaras Hindu University, Varanasi, India; ^2^Centre for Bioinformatics, School of Biotechnology, Institute of Science, Banaras Hindu University, Varanasi, India; ^3^Division of Crop Improvement and Biotechnology, Indian Institute of Vegetable Research, Indian Council of Agricultural Research (ICAR), Varanasi, India; ^4^Bioinformatics Centre, Department of Bioinformatics, Sri Venkateswara Institute of Medical Sciences University, Tirupati, India; ^5^Department of Botany, University College of Science, Mohanlal Sukhadia University, Udaipur, India

**Keywords:** THN reductase, fungicide, melanin, protein–fungicide interaction, homology modeling, MD simulations, MM/GBSA analysis

## Abstract

Vascular wilt of tomato caused by *Fusarium oxysporum* f.sp. *lycopersici* (FOL) is one of the most devastating diseases, that delimits the tomato production worldwide. Fungal short-chain dehydrogenases/reductases (SDRs) are NADP(H) dependent oxidoreductases, having shared motifs and common functional mechanism, have been demonstrated as biochemical targets for commercial fungicides. The 1,3,6,8 tetra hydroxynaphthalene reductase (T4HNR) protein, a member of SDRs family, catalyzes the naphthol reduction reaction in fungal melanin biosynthesis. We retrieved an orthologous member of T4HNR, (complexed with NADP(H) and pyroquilon from *Magnaporthe grisea*) in the FOL (namely; FOXG_04696) based on homology search, percent identity and sequence similarity (93% query cover; 49% identity). The hypothetical protein FOXG_04696 (T4HNR like) had conserved T-G-X-X-X-G-X-G motif (cofactor binding site) at N-terminus, similar to *M. grisea* (1JA9) and Y-X-X-X-K motif, as a part of the active site, bearing homologies with two fungal keto reductases T4HNR (*M. grisea*) and 17-β-hydroxysteroid dehydrogenase from *Curvularia lunata* (teleomorph: *Cochliobolus lunatus* PDB ID: 3IS3). The catalytic tetrad of T4HNR was replaced with ASN^115^, SER^141^, TYR^154^, and LYS^158^ in the FOXG_04696. The structural alignment and superposition of FOXG_04696 over the template proteins (3IS3 and 1JA9) revealed minimum RMSD deviations of the C alpha atomic coordinates, and therefore, had structural conservation. The best protein model (FOXG_04696) was docked with 37 fungicides, to evaluate their binding affinities. The Glide XP and YASARA docked complexes showed discrepancies in results, for scoring and ranking the binding affinities of fungicides. The docked complexes were further refined and rescored from their docked poses through 50 ns long MD simulations, and binding free energies (ΔG_bind_) calculations, using MM/GBSA analysis, revealed Oxathiapiprolin and Famoxadone as better fungicides among the selected one. However, Famoxadone had better interaction of the docked residues, with best protein ligand contacts, minimum RMSD (high accuracy of the docking pose) and RMSF (structural integrity and conformational flexibility of docking) at the specified docking site. The Famoxadone was found to be acceptable based on *in silico* toxicity and *in vitro* growth inhibition assessment. We conclude that the FOXG_04696, could be employed as a novel candidate protein, for structure-based design, and screening of target fungicides against the FOL pathogen.

## Introduction

Tomato (*Lycopersicon esculentum* Mill.) is one of the most widespread vegetable crops grown across the globe. However, the growth and economic productivity of tomato crop are well constrained by various biotic and abiotic stress conditions (Bergougnoux, 2014; Gupta and Rashotte, 2014). Vascular wilt disease caused by *Fusarium oxysporum* f.sp. *lycopersici* (FOL) (Sacc.) W. C. Snyder and H. N. Hans (FOL) is one of the most destructive diseases (Amini and Sidovich, 2010; Prihatna et al., 2018), that affects the growth and economic production of tomato (Yeole et al., 2016; Prihatna et al., 2018). The wilt pathogen FOL is the most common soil-borne Ascomycetous fungus that infects through roots and develops symptoms leading to vascular wilt in tomato (Park et al., 2013; Rongai et al., 2017). It invades the xylem vessels resulting in wilting and death of the plant (Swarupa et al., 2014). The high-frequency incidence (25–55%) of *Fusarium* wilt disease in tomato has been reported from various regions of India (Asha et al., 2011; Pandey and Gupta, 2014; Nirmaladevi et al., 2016). The infection and disease development of the fungus leads to devastating agricultural losses, which may cover up to 80% under the favorable weather conditions.

The vascular wilt disease of tomato is characterized by vascular browning, that involves the deposition of melanin-like compounds on the walls of xylem vessel and other neighboring parenchymatous cells (Mace et al., 2012). The control of vascular wilt disease is difficult and mainly achieved through the use of chemical fungicides (Minton, 1986; DeVay et al., 1988; Swarupa et al., 2014). The most commonly used chemical fungicides that have been used up to till date against the *Fusarium* sp. either alone or in combination with other integrated approaches includes iprodione (Amany and Ellil, 2005) (Rovral) (dithiocarboxamide) benomyl (Benelate) carbendazim, prochloraz, fludioxonil, bromuconazole, azoxystrobin (Amini and Sidovich, 2010; Singha et al., 2011; Anand et al., 2013; Khan et al., 2014), flutolanil (Moncut WP 30%), tolclofos-methyl/thiram (Rhizolex 50% WP) and carboxin-thiram (Vitavax 200 WP) (Mohamed and Amer, 2014), mancozeb + carbendazim (0.125 + 0.05%) (Barhate et al., 2015), mancozeb + copper sulfate + copper oxychloride (Ramaiah and Garampalli, 2015), metiram (55%) and pyraclostrobin (5%) (Yeole et al., 2016), thiophanate methyl (La Torre et al., 2016), propiconazole, thiabendazole, benomyl, fuberidazole, thiophanate, myclobutanil triadimefon, difenoconazole, tebuconazole, epoxiconazole, methoxy-acrylates, ethyl phosphonates (de la Isla and Macías-Sánchez, 2017), Nativo 75% WG, Cordate 4WP, fluopyram 20% + tebuconazole 20%, and tebuconazole 50% + trifloxystrobin 50% (Patón et al., 2017).

Short-chain dehydrogenases/reductases (SDRs) are NADP(H)-dependent oxidoreductases characterized by conserved catalytic tetrad (N-S-Y-K) and cofactor binding site (TGxxxGxG) (Jörnvall et al., 1995; Filling et al., 2002) with having common α/β-folding pattern, and characterized by presence of a central β-sheet typical to Rossmann-fold with helices on either side (Kavanagh et al., 2008). The fungal 1,3,6,8-tetrahydroxynaphthalene reductase belongs to SDR family mediates the naphthol reduction reactions in melanin biosynthetic pathway (Liao et al., 2001). The protein Blast results at NCBI revealed that *M. grisea* T4HNR (SDR) showed high sequence similarity with other fungal keto reductases, involved in the biosynthesis of fungal melanin and mycotoxins, that includes versicolorin reductase from *Magnaporthe oryzae* (99%), *Verticillium alfalfae* (77%), *Verticillium dahliae* (76%), *Colletotrichum graminicola* (79%), versicolorin reductase (VerA) from *Emericella nidulans* (52%), and 17β-hydroxysteroid dehydrogenase (17β-HSD*cl*) of *Cochliobolus lunatus* (52%). The crucial role of the fungal SDR gene in *M. oryzae* is required for infection related development and pathogenicity (Kwon et al., 2010). The function of a novel fungal SDR gene (*adh1*) encoding for alcohol dehydrogenase has been reported to play a crucial role in virulence of Fusarium wilt pathogen in tomato (Corrales et al., 2011).

**FIGURE 1:**
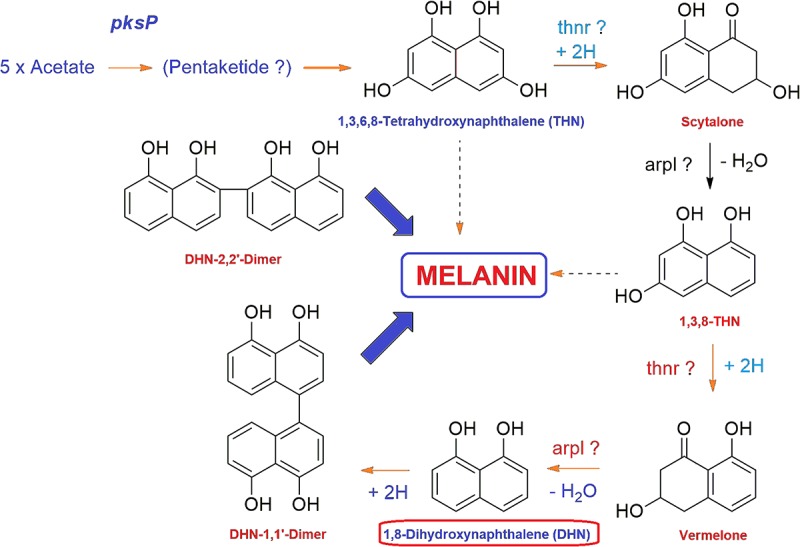
General mechanism of DHN melanin biosynthesis pathway in fungi. The tetrahydroxynaphthalene reductase (T4HNR) catalyzes the NADP(H)-dependent reduction of 1,3,6,8-tetrahydroxynaphthalene (THN) into (+)-scytalone and 1,3,8-trihydroxynaphthalene into (–)-vermelone. 1,8-dihydroxynaphthalene (DHN) is the immediate precursor of the polymer.

Fungal melanins are high molecular weight dark brown to black colored pigments synthesized via the pentaketide pathways in the cell wall of fungal groups belonging to Ascomycotina and Deuteromycotina (Bell and Wheeler, 1986). The DHN melanin biosynthetic route is the most common among fungi where melanins are synthesized through the acetate via the polyketide synthase pathway (Chiewchanvit et al., 2017). DHN melanin pathway has been investigated in many filamentous plant pathogenic fungi including *Cochliobolus heterostrophus* (Eliahu et al., 2007), *Alternaria* spp. (Kheder et al., 2012), *Colletotrichum* spp. (Ludwig et al., 2014), genera *Gaeumannomyces* (Frederick et al., 1999), *Phyllosticta musarum* (Kubo and Furusawa, 1991), and *V. dahliae* (Wheeler et al., 1978). During the biosynthesis of fungal melanin through pentaketide pathway, tetrahydroxynaphthalene reductase (T4HNR) catalyzes the NADP(H)-dependent reduction of 1,3,6,8-tetrahydroxynaphthalene (THN) into (+)-scytalone and 1,3,8-trihydroxynaphthalene into (−)-vermelone (Figure 1). The DHN pathway-based classification depends on their preference for the Naphthoquinon precursors or on the effect of inhibitors such as phthalide or tricyclazole, which binds with hydroxynaphthalene reductases having classical short-chain dehydrogenase/reductase (SDR) with Rossmann-fold domains (Palonen et al., 2017). It has been reported that the polyketide pathway in filamentous fungi is an important metabolic process that regulates their growth, development, and pathogenicity (Xiong et al., 2014). Fungal melanin is an important polyketide and genes responsible for the biosynthesis of melanins have been reported in *V. dahliae* including hydroxynaphthalene reductase (*VDAG_03665*), polyketide synthase (*VDAG_00190*), and scytalone dehydratase (*VDAG_03393*) (Xiong et al., 2014). Recently, the gene clusters and enzymes, involved in melanin and other pigment biosynthesis, were explored in Ascomycota including *Aspergillus* spp. based on transcriptomic and gene expression studies. The studies revealed that the core polyketide synthase (PKS) gene clusters have crucial role in biosynthesis of DHN type of pigment (Palonen et al., 2017). The phylogenetic analysis of the extended PKS revealed striking similarities with group of known pigments of *Fusarium* spp., which predicts the similar function for this PKS (Palonen et al., 2017). Some chemical fungicides that inhibit the biosynthesis of melanin have been used in controlling plant pathogenic fungi (Kurahashi, 2001). In the last few years, many melanin biosynthesis inhibitors have been used against rice blast pathogen such as triazoloquinoline, pyroquilon, tricyclazole, and coumarin (Yamaguchi and Kubo, 1992; Kimura and Tsuge, 1993). The formation of melanin by the members of *Fusarium* genus has been recently reported as it was found that *F. graminearum* accumulates melanins in a process dependent on polyketide synthase PGL1 (Frandsen et al., 2016). Furthermore, *F. keratoplasticum*, a significant causing agent of fusariosis produces melanin or melanin-like compounds during *in vitro* cultivation and also inside the growing tissues as confirmed through immunofluorescence labeling with anti-melanin monoclonal antibody (MAb) (Chiewchanvit et al., 2017). The fungus FOL forms brown colored melanin that is insoluble in water and organic solvents but soluble in alkaline medium (1 M KOH) (Amany and Ellil, 2005). Dicarboxamide produces antimicrobial oxidants using ROS molecules, thus inhibiting the growth of many pathogenic fungi, including *F. oxysporum* (Abo Ellil and Sharaf, 2000). The sensitivity of some potent phytopathogenic fungi such as *Sclerotium cepivorum*, *Alternaria alternata*, and FOL pathogen against melanin biosynthesis inhibitor (fungicides having dicarboxamide group) have been well evaluated (Amany and Ellil, 2005). Furthermore, fungicides that inhibit the biosynthesis of melanin (tricyclazole, pyroquilon, and iprodione) could be employed as a useful tool for controlling plant pathogenic fungi that utilize polyketide metabolites as intermediates (Motoyama and Yamaguchi, 2003; Singh et al., 2014). The 17-β-hydroxysteroid dehydrogenase (SDR) was recently used as a molecular target for fungicide tricyclazole against *Cercospora canescens*, causing Leaf spot disease in mung bean (*Vigna radita*) (Singh et al., 2014). In a recent study, the inhibitors for *F. oxysporum* copper nitrite reductase (NirK), involved in the fungal denitrification process were searched using hierarchical *in silico* screening approach that consists of pharmacophore modeling and molecular docking (Matsuoka et al., 2017). The ranges of the molecular target for currently used fungicides are narrow, and therefore, the threat of resistance development necessitates the need for the discovery of novel targets for fungicides (Foster, 2018).

In the last few years, several studies have been done on *in silico* characterization of an unknown hypothetical proteins/essential genes from pathogenic microbes, that might have a possible role in regulation of metabolic process, or play an indispensable role in microbial pathogenicity (Ravooru et al., 2014; Silva et al., 2015; Marklevitz and Harris, 2016; Kumar et al., 2017; Prava et al., 2018). Recently, a hypothetical protein (FcRav2) with ROGDI such as leucine zipper domain, and homologous to yeast Rav2 was reported in *F. culmorum*. It was demonstrated that FcRav2 protein may become a suitable target for new antifungal drug development or the plant–mediated resistance response in filamentous fungi of agricultural interest (Spanu et al., 2018).

In this study, we have predicted and characterized a fungal SDR (the FOXG_04696) as a putative receptor protein, and a novel target, for structure-based protein–fungicide complex interactions. The predicted protein was found to be good enough based on qualitative and quantitative parameters and was further docked with 37 known commercial fungicides, frequently used against different phytopathogens, to find the best fungicide/agrochemicals (among the selected) that could target the FOXG_04696 and therefore, useful for controlling vascular wilt fungi. The environmental toxicity assessment could be used to decide the dosage formulations that could be used safely without having any loss to the non-target organism. With this view, the selected fungicides were further evaluated based on *in silico* toxicity assessment tools. It was found the fungicide that binds with crucial residues forming active site of the receptor protein (disrupt the protein function) have a better fungicidal action [for example, T4HNR complex with pyroquilon and NADP(H) used successfully against *M. grisea*] (Singh et al., 2014). The objective of the present study is to evaluate the efficacy of potential inhibitor (fungicides) that could bind to the crucial residues of the FOXG_04696. Furthermore, the protein–fungicide docking studies with target protein could be useful to evaluate the comparative efficacy of an individual fungicide over each other against vascular wilt pathogen.

## Materials and Methods

### Database Search, Comparative Phylogeny, and Functional Domain Analysis

The protein sequence available for the crystal structure of the T4HNR protein complexed with NADP(H) and pyroquilon fungicide, and solved through X-ray diffraction in *Magnaporthe grisea* was selected for searching all the sequential homolog and orthologs using NCBI Blast server^1^ (Altschul et al., 1997) keeping the default values, and against the non-redundant protein sequences, with searching the organism as FOL 4287 (taxid: 426428). The sequences were also retrieved, checked, and confirmed from the JGI genome portal for FOL with having transcript ID 13950. The Blast-p annotations were further checked across several databases. The FOXG_04696 homolog and orthologous sequences to the T4HNR protein were identified using Blast-p and collected for multiple sequence alignment using ClustalW (Thompson et al., 1994). Multiple sequence alignment was done to represent the consensus and conserved residues present in the T4HNR protein across the different members using CLC BIO workbench. The alignment results were further checked using the BioEdit tool (Hall, 1999). The phylogenetic relationship between the different homolog and orthologs were established using the neighbor-joining (NJ) and maximum parsimonious method using the MEGA6 suite^2^ (Tamura et al., 2013) at 1000 replication bootstrap values. The similarities and differences in the T4HNR proteins in between different homologous and orthologous fungal partners were visualized based on the comparison of their protein sequences retrieved through the conservation of genomic positions (segments) using circos visualization tool^3^ (Krzywinski et al., 2009) at 50% cutoff filter values. The functional domain of the identified protein was searched using ExPASy-PROSITE scan^4^ (de Castro et al., 2006; Sigrist et al., 2010). The identified FOXG_04696 protein sequences were further searched for finding the functional signature sequences against the InterPro protein signature database using InterProScan 5.0^5^ (Jones et al., 2014).

### Gene Prediction and Chromosomal Mapping

Sequence of the protein tetrahydroxynaphthalene reductase (T4HNR) complexed with NADP(H) and pyroquilon (1JA9) was searched to find its sequential orthologs in the FOL pathogen, using Blast-p against the non-redundant database. Furthermore, the two protein sequences (1JA9 and the FOXG_04696) were aligned using BL2 seq (Blast-p). The FOXG_04696 protein sequence was also aligned with the protein sequence of 17-β-hydroxysteroid dehydrogenase [other closely related structural homolog (3IS3)]. Furthermore, the FOXG_04696 protein was searched using the tBlastn against the Refseq (reference protein) genome database, searching for *F. oxysporum* f.sp. *lycopersici*. The first hit obtained was further scanned with the gene prediction tool Fgenesh^6^. Furthermore, the chromosomal map was generated to identify and locate the position of the gene, that encodes the hypothetical protein FOXG_04696 using the Ensembl-BLAST tool.

### CATH Analysis

The functional annotation of the predicted FOXG_04696 protein was done using CATH server. The FOXG_04696 protein sequence was submitted to CATH database^7^ (Sillitoe et al., 2015) for structural classification, based on domains organization, and folding patterns that belong to homologous protein superfamilies. The FunFHMMer^8^ (Das et al., 2016) was used for functional classification of the identified CATH super families. The ReviGO webserver^9^ (Supek et al., 2011) was used for plotting the functional annotation in terms of molecular function and biological processes involved using scattered plot diagram. The CELLO2GO webserver^10^ (Yu et al., 2014) was used for finding the probable subcellular localization of the predicted protein. The possible functional role of the FOXG_04696 was predicted in terms of gene ontology enrichment analysis.

### Identification of Functional Sites

The functional sites of the identified protein were searched using CD search on CDD webserver^11^ (Marchler-Bauer et al., 2015, 2017) at three interfaces including protein active site, substrate binding site, and chemical binding (NADP binding site). The meta-pocket server^12^ (Huang, 2009) was used for the prediction of three prominent binding sites in the FOXG_04696 protein.

### Protein–Protein Interaction Network

The FOXG_04696 protein was submitted to the STRING (Search Tool for the Retrieval of Interacting Genes/Proteins database version 10.0)^13^ (Szklarczyk et al., 2007) server for the functional interaction associative network between the partners, and the interactions were analyzed at their high and highest confidence level.

### Structural Modeling

The homology modeling of the protein FOXG_04696 (T4HNR like) was performed using Modeller v9.19. The protein sequence was queried against the PDB database^14^ (Berman et al., 2000) with having sequence similarities >90% using Blast-p to identify the closely related structural homologs for the FOXG_04696. The first hit obtained on Blast-p annotation was found to 17-β-hydroxysteroid dehydrogenase (SDR enzyme) from *Cochliobolus lunatus* was taken as a template (PDB ID: 3IS3; 46% identity, 96% of query coverage; E-value of 2e–75). The PDB file of the template (3IS3) was retrieved from the Protein Data Bank (PDB). The alignment file was generated using CLUSTALX. The target sequence file, alignment file, and template’s PDB file, PDB file (3IS3) was initialized in the Modeller script file (script.py). The script file (script.py) was executed using Modeller command prompt. Twenty-five models were generated for the FOXG_04696, each with having a DOPE score. Furthermore, the protein model with least DOPE score was selected for final validation.

### Model Validation

The stereochemical stability of the predicted models were further verified using various protein quality based parameters such as percentage residues lying in favored and allowed regions, the number of glycine and proline residues and orientation of dihedral angles including phi (φ) and psi (*ψ*) and backbone conformation using PROCHECK module of the PDBSum server^15^ (Laskowski et al., 2005), and also confirmed using the RAMPAGE server^16^ (Lovell et al., 2003). The qualitative assessment methods were based on ProSA analysis (probable residues lying at a specific distance and interactions observed between the model and the solvent i.e., solvation^17^ (Wiederstein and Sipp, 2007). The VERIFY3D (Eisenberg et al., 1997) server was used to check the compatibility of atomic models (3D) with its own primary amino acid sequences (1D). The quality was verified using the ERRAT score values^18^ (statistics of non-bonded atomic interactions and distribution of atoms) (Colovos and Yeates, 1993). The overall quality assessment of predicted model was done through ProTSAV score values^19^ (Singh et al., 2016). The quantitative evaluation of the model was done through the VADAR^20^ (Willard et al., 2003). The modeled FOXG_04696 protein was superimposed over the template T4HNR (SDR) protein of *M. grisea* (1JA9) to compare their structural alignment and similarities using the Automated Structural Alignment Server (AuStrAlis)^21^. The final model was submitted to an online repository protein modeling databases (PMDB)^22^ (Castrignano et al., 2006).

### Preparation of Protein and Ligands

The ligands were retrieved from the PubChem database. The FOXG_04696 protein was selected as a target receptor protein and was imported to the Maestro v11. The structure was prepared using protein preparation wizard of the Schrödinger. Optimization of protein was done at neutral pH and then the structure was minimized by applying optimized potentials for liquid simulations (OPLS-3) force field for all atoms (Umamaheswari et al., 2010). A receptor grid of 10Å × 10Å × 10Å was generated on defined binding site residues of the FOXG_04696 using Glide v7.1 (Grid-based Ligand Docking with Energetics, Schrödinger, LLC, New York, NY, United States, 2017) (Friesner et al., 2006). The ligand was prepared through adjusting the chemical correctness (protonation), stereochemical and ionization variation using Epik and LigPrep modules. The energy minimization was done at neutral pH 7.0 ± 2.0.

### Protein–Fungicide Docking

The Glide XP ligand docking protocol was employed to predict the scoring and binding interactions between the FOXG_04696 and the ligand Famoxadone. The prepared ligand was docked into the binding site of the FOXG_04696. The van der Waals radii of non-polar regions of the T4HNR were limited to 1.0Å with the partial atomic charge of 0.25 in the receptor grid generation. Rigid receptor docking was utilized to dock each ligand into every refined low-energy conformation of the T4HNR produced from the earlier phases (high-throughput virtual screening and standard precision methods). XP docked complexes were evaluated using Xtra precision Glide score (XPG Score). The XPG score optimized the ligand binding energy on the behalf of the force field parameters, and penalties that had significant influences over the receptor-ligand binding. The following equation denotes the formulae for XPG calculations.

$$\begin{array}{ccc} \mathrm{Score} & = & \;a*\mathrm{vdW}+b*\mathrm{Coul}+\mathrm{Lipo}+\mathrm{Hbond}+\mathrm{Metal}\\ & & +\mathrm{BuryP}+\mathrm{RotB}+\mathrm{Site} \end{array}$$

where vdW, Coul, Lipo, H bond, metal, BuryP, Rot B, and Site denote van der Waals energy, Coulomb energy, lipophilic contacts, hydrogen-bonding, metal-binding, penalty for buried polar groups, penalty for freezing the rotatable bonds, and polar interactions with the residues in the active site, respectively; *a* = 0.065 and *b* = 0.130 are coefficient constants of van der Waals energy and Coulomb energy, respectively.

The molecular docking of the FOXG_04696 with fungicides was also performed through YASARA (Yet Another Scientific Artificial Reality Application) (Krieger and Vriend, 2014; Chen et al., 2015). The YASARA docked protein–fungicide complexes were analyzed for the comparative binding energies and dissociation constant (*K*_d_) of the docked molecular complexes (Yadav et al., 2017).

### Molecular Mechanics and Binding Energy Assessment

The protein–fungicide docked complexes were further analyzed for Molecular Mechanics/Generalized Born Surface Area (MM/GBSA) analysis to predict the free binding energies of the protein–fungicide docked complexes. The binding energy calculated through MM/GBSA was more accurate than the XPG _Score_ (Lyne et al., 2006). The binding free energy Δ*G*_bind_ was calculated by the following equations (Liang et al., 2017; Zhang et al., 2017).

$$\begin{array}{ccc} \Delta G_{\mathrm{bind}} & = & \Delta G_{\mathrm{complex}}-(\Delta G_{\mathrm{receptor}}+\Delta G_{\mathrm{ligand}})\\ \Delta G & = & \Delta E_{\mathrm{gas}}+\Delta G_{\mathrm{sol}}-T\Delta S_{\mathrm{gas}}\\ \Delta E_{\mathrm{gas}} & = & \Delta E_{\mathrm{int}}+\Delta E_{\mathrm{ELE}}+\Delta E_{\mathrm{VDW}}\\ \Delta G_{\mathrm{sol}} & = & \Delta G_{\mathrm{GB}}+\Delta G_{\mathrm{Surf}} \end{array}$$

These energy contributions are computed from the atomic coordinates of the protein, ligand and complex using the (gas phase) molecular mechanics energy function (or force field). The solvation free energy term *G*_solv_ contains both polar and non-polar contributions. The binding free energy (Δ*G*_bind_) could be dissociated into various energy terms. Since the same trajectory was selected for extraction of receptor protein, ligand, and protein–ligand complex, we neglected the internal energy change (Δ*E*_int_). Therefore, the gas–phase interaction energy (Δ*E*_gas_) between the receptor and the ligand was the sum of electrostatic (Δ*E*_ELE_) and van der Waals (Δ*E*_V DW_) interaction energies. The solvation free energy (Δ*G*_sol_) could be distributed into non-polar and polar energy terms, and the polar solvation energy (Δ*G*_GB_) is calculated by using the VSGB2.1 GB model, and was default parameter for Prime calculations using the OPLS2.1/3/3e force field. The Post-docking MM/GBSA is implemented in Schrödinger software using the program Prime, with options to include receptor and ligand flexibility; the entropy term is neglected by default. Simulations were performed using GBSA continuum model. The Gaussian surface area model instead of vdW was employed for denoting the solvent accessible surface area.

### Molecular Dynamics (MD) Simulations

The receptor–ligand interactions for fungicides having minimum binding energy (stronger binding) were further evaluated using molecular dynamics simulations analysis. MD simulations studies were performed up to 50 ns through Desmond v 4.2 to analyze the conformational stability of the FOXG_04696–Famoxadone, and the complexes in the solvated model system, embedded with ordered water molecules (ordered water molecules may involve in protein binding sites and influence protein ligand binding by bridging protein–ligand interactions and can make large contributions to the binding affinity). The Desmond supports algorithms typically used to perform fast and accurate MD simulations. Long-range electrostatic energy and forces were calculated using particle-mesh-based Ewald techniques. The FOXG_04696–ligand docked complexes were solvated, using orthorhombic simple point charge (SPC) water model. The solvated system was neutralized with counter ions and physiological salt concentration was limited to 0.15 M. The receptor–ligand complex system was assigned with optimized potentials for liquid simulations-AA (OPLS-AA) 2005 force field (Madhulitha et al., 2017). The system was specified on periodic boundary conditions, the particle mesh Ewald (PME) (Maragakis et al., 2008) method was applied for electrostatics. Lennard-Jones interactions cutoff was set to 10Å and SHAKE algorithm (Friesner et al., 2006) was employed for limiting movement of all covalent bonds involving hydrogen atoms. The solvated model system, prior to MD simulationss study, was passed through a six-step relaxation protocol for energy minimization (Katari et al., 2016). At first, only solvent molecules were allowed for energy minimization which then followed by minimization of the entire system using the Broyden–Fletcher–Goldfarb–Shanno (LBFGS) algorithm (Chiranjeevi et al., 2016). The minimized system was further analyzed with NVT ensemble for 12 picoseconds (ps) simulationss at 10 K temperature. The non-hydrogen solute atoms were restrained at 300 K temperature for 24 ps. Furthermore, the system was simulated for 24 ps in the NPT ensemble at 300 K temperature without restrains in order to attain an equilibrium state (Chubb et al., 2006). The minimized system without any restrains was further subjected to 50 ns NPT simulations production (Cichero et al., 2013; D’Ursi et al., 2016). Berendsen thermostats and barostat were used to control the temperatures and pressures during the initial simulations (Pradeep et al., 2015). For MD simulations, Desmond was utilized as constraints, which are enforced using a variant of the SHAKE algorithm, allowed the time step to be increased. These approaches can be used in combination with time-scale splitting (RESPA-based) integration schemes. The purpose was to find the interactions between protein and ligand in protein–ligand complex during MD simulations.

### *In vitro* Inhibition Test

The selected fungicide (Famoxadone) was used for *in vitro* assessment against the FOL pathogen. The pathogenic culture was obtained from Laboratory of Mycopathology and Microbial Technology, Department of Botany, Institute of Science, Banaras Hindu University, Varanasi, India and the fungicide Famoxadone (Sigma-Aldrich, St Louis, MO, United States) was used for evaluating its *in vitro* efficacy. Four separate concentrations 50, 100, 150, and 200 μL were employed along with the control solution (having only PDA) and amended in 20-mL PDA medium. A 5-mm culture disc was extracted from the freshly inoculated pathogen culture in each of the four plates. The plates were further incubated at 27 ± 2°C under observation and the radial growth of the hyphae was recorded at even (2, 4, 6, and 8) days interval.

The percent growth inhibition (PI) was calculated using the following formula [(*C* − *T*)/*C* × 100] where *I* = inhibition percentage; *C* = radial growth of the pathogen in control, and *T* = radial growth of the pathogen fungicide treatment (Suneeta et al., 2016). The percentage inhibitions measured in the form of radial growth were subjected to statistical analysis. All the experiments were executed in triplicates and repeated twice employing a completely randomized design. The representative statistical data were expressed in mean ± SEM values of three independent replications data ± SD, and the average data of one experiment was interpreted through one-way analysis of variance (ANOVA), while the comparison of mean separations was performed with Duncan’s multiple range test (DMRT) with *P* ≤ 0.05 of significance level.

### *In silico* Toxicity Assessment

The *in silico* toxicity assessment of the selected fungicide was made with different online tools and software including FAF-Drugs 4.0^23^ (Lagorce et al., 2017). Furthermore, the environmental toxicity hazard assessments were also evaluated through admetSAR^24^ (Cheng et al., 2012). The drug-likeness of the selected fungicide was evaluated through Lipinski Rule of Five using the server given in the web link^25^ (Lipinski, 2004; Jayaram et al., 2013).

## Results

### Database Search, Comparative Phylogeny, and Functional Domain Analysis

**FIGURE 2:**
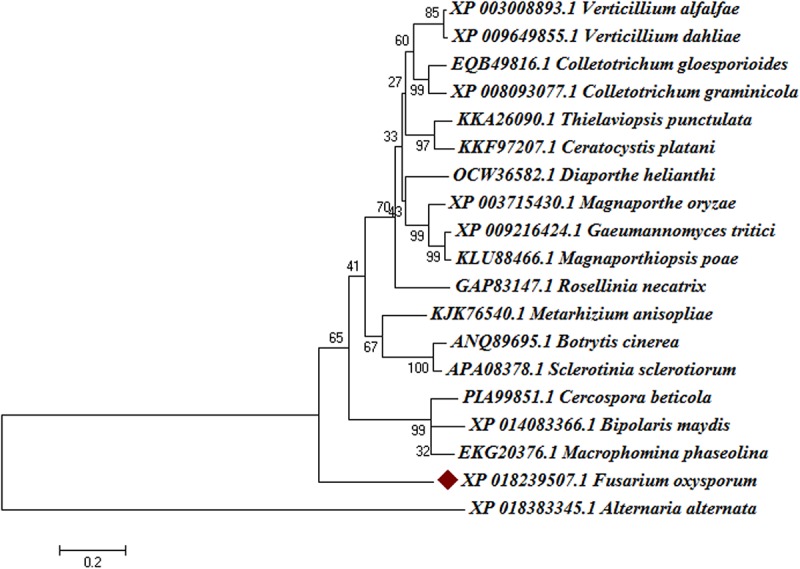
Phylogenetic relationships between the different fungal taxa showing the evolution of short-chain dehydrogense/reductases (T4HNR like) protein. The tree was constructed based on distance-based neighbour-joining (NJ) method with 1000 bootstrap relications using MEGA6.0. The tree showed the existence of several clades for fungal short-chain dehydrogenases/reductases (SDRs) between the evolutionarily related taxa. The hypothetical protein (FOXG_04696) lacks common ancestor and therefore predicts the polyphyletic evolution of SDR in *Fusarium oxysporum* f.sp. *lycopersici*. The bootstrap values are mentioned below the tree.

**FIGURE 3:**
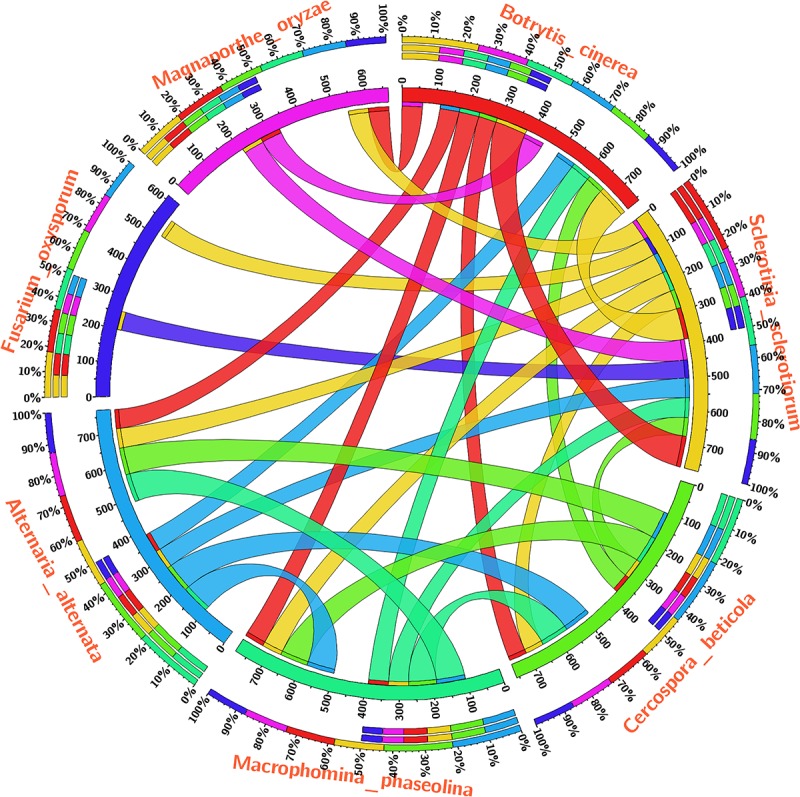
The circos visualization map showing the similarities and differences for the SDRs (T4HNR like) among the five major phytopathogenic fungi, retrieved from genome comparision (based on sequential alignment). The circos map was generated at 50% cutoff score values and drawn using percentage identity matrices, calculated and obtained during phylogenetic clustering of the protein sequences using ClustalW, and represented the positional conservation and relationship between the genomic intervals.

The Blast-p results against the non-redundant (nr) database with organism *Fusarium oxysporum* f.sp. *lycopersici* 4287 (taxid: 426428) revealed the homology of the query protein sequence (1JA9) with the target protein FOXG_04696. The query sequences showed 93% query coverages with 49% identity with the target protein FOXG_04696 (XP_018239507.1). The PDB Blast-p annotation revealed the queried sequence of the FOXG_04696 had more than one structural homologs like 1JA9 (47% identity), 3IS3 (44% identity), and therefore, could be used as a template protein for homology modeling of our target protein. The Uniprot results identified the queried protein sequence as an uncharacterized/hypothetical protein of *F. oxysporum* f.sp. *lycopersici* (strain 4287/CBS 123668/FGSC 9935/NRRL 34936) (A0A0D2XL72). Interestingly, both T4HNR (1JA9) and 17-β-hydroxysteroid dehydrogenase (3IS3) query sequences when searched against the reference protein (Ref seq) database, with searching for *Fusarium* (taxid: 5506) the first and significant hit obtained showed an orthologous relationship of the queried protein with the hypothetical protein FOXG_04696 [XP_018239507.1; 49% identity (1JA9): 93% query coverages; E-value: 3e–74 and 46% identity (3IS3): 94% query cover; E-value: 1e–71], which further confirms the existence of similar T4HNR and 17-β-hydroxysteroid dehydrogenase-like protein (FOXG_04696) in the FOL pathogen. The phylogenetic tree was constructed based on the neighbour-joining end (NJ) method revealed the polyphyletic origin of the FOXG_04696 protein (Figure 2). The evolutionary conservation and functional diversification of the fungal SDRs across the related taxonomic group have been shown through maximum parsimonious method based phylogenetic tree (Supplementary Figure S1). The PROSITE results revealed the presence of common functional domain with signature sequences characteristic to the SDR family (IPR002347) and the NADP binding domain superfamily (InterproID: IPR036291) (Supplementary Figure S2). The circos results revealed the polyphyletic ancestry of the FOL with other Ascomycetous fungal taxa at highest filter cutoff values. However, at the medium scale (50% cutoff score) we found similarity index at their low percentage values, with the other homolog and orthologous members (Figure 3). The multiple sequence alignment results showed the strong conservation of core residues (red square) occupied within the functional domain, with the substitution of some residues at consensus positions (Supplementary Figure S3). It has been reported that the aldo–keto reductase superfamily might have been evolutionarily diverged from an ancestral multifunctional oxidoreductases (Jez et al., 1997). However, the presence of similar and identical active sites, across the distantly related fungal taxonomic group, explained their convergent evolution as SDRs superfamily (Jez et al., 1997). The conserved domain database alignment results for the queried protein identified the conserved functional sites that include (both active site and substrate binding site) across the evolutionary diverged fungal partners.

### Gene Prediction and Chromosomal Mapping

**FIGURE 4:**
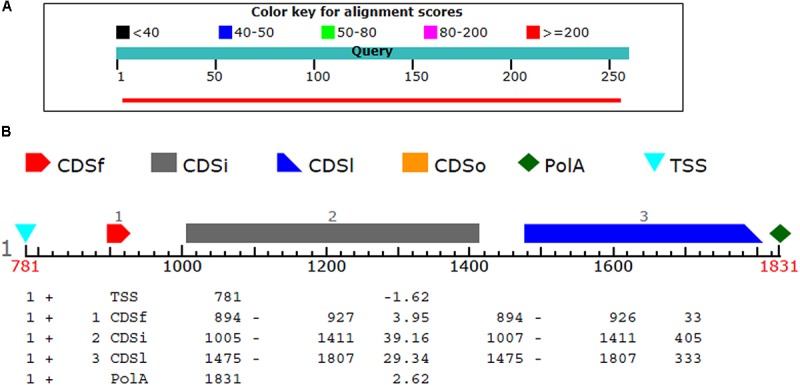
**(A)** The comparison of the query protein (FOXG-04696) with the protein sequences of the T4HNR (*M. grisea*). The two protein sequences (1JA9 with the FOXG-04696) were aligned using the BL2seq (Blast-p). The hypothetical protein FOXG_04696 show more sequence similarity based on the percentage identities (49%) and query coverages (97%) with the 1JA9. The other structural homolog the 17-β-hydroxysteroid dehydrogenase (3IS3) had percentage identities (46%) and query coverages (96%). **(B)** Prediction of the FOXG_04696 protein encoding gene with coding sequences, transcription start sites and Poly A tail.

The BL2seq (Blast-p) results revealed that (XP_018239507.1) protein was found to have (97% query coverages; 50% identity; E-value 3e–78) with the T4HNR protein of *M. grisea* (XP_003715430.1). By contrast, the BL2seq query with 3IS3 resulted into (94% query coverages; 46% identity; E-value 8e–77). This confirms that T4HNR (1JA9) is closely related with 1JA9 based on percentage identity and query cover values. The Fgenesh results located the position of the FOXG_04696 encoding gene along with transcriptional start sites (TSS) and poly A tail across the full-length genome (Figure 4). The FOXG_04696 gene was found to be located on chromosome7 (NC_030992.1 with 87% identity; 99% query coverages; E-value 2e–150). The chromosomal map represented the position of the gene (FOXG_04696) on chromosome 7 (genomic bp 7: 22061–22974; 100% identity; E-value 0.0) (Supplementary Figure S4).

### Structural Modeling, *in silico* Characterization, and Model Validation

**FIGURE 5:**
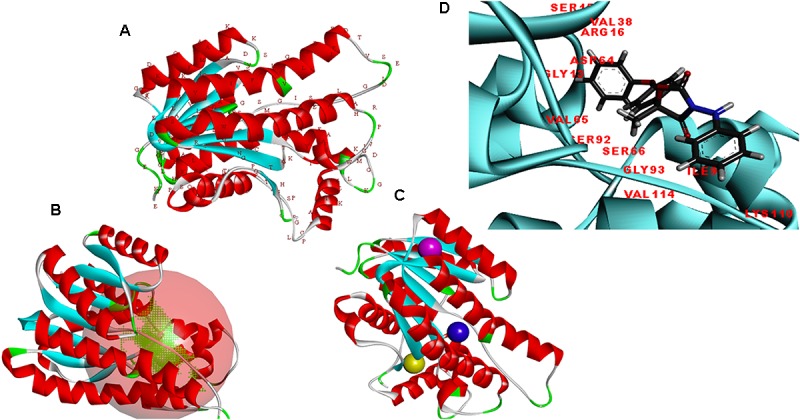
**(A)** Predicted structure of the FOXG_04696 modeled through homology modeling using Modeller v9.19 and visualized through the Discovery Studio 3.0 visualization tool. **(B)** The big red sphere represents the cavities surrounding the active sites and was visualized using the visualization module of the Discovery studio 3.0. The three binding sites were explored through the meta-pocket server. **(C)** The three putative binding sites as shown through three different colored red balls. **(D)** General view of protein-ligand interaction showing the residues from the active site (FOXG_04696) residues involved in making interaction with ligand (fungicide).

The modeler generated 25 predictive models for protein FOXG_04696 with different discrete optimized potential energy (DOPE) score values. The model with least values for DOPE score (21st model; −28563.03 kcal/mol) was selected as a final model for *in silico* characterization and docking studies. The predicted model was visualized through the visualization module of the Discovery Studio 3.0 (Figure 5A). The three putative prominent binding sites identified in the target protein structure have been shown (Supplementary Table S1). It was found that most of the residues involved in binding to ligand (fungicide) were occupied from first the major binding, site (binding site 1; metapocket results) of the predicted model. The major catalytic sites inside the protein occupying all the potential residues that get involved in binding with ligands have been shown in Figure 5B with red balls showing active sites (Figure 5C). The residues that constitute the functional ligand-binding sites have been shown (Figure 5D).

**FIGURE 6:**
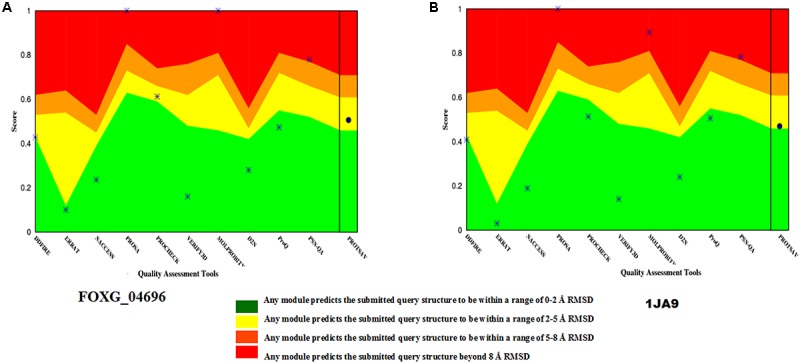
The qualitative assessment of the predicted model FOXG_04696 and its comparative evaluation with the X-Ray resoluted template proteins (1JA9) using the ProtSAV score. **(A)** The qualitative assessment of the modelled protein (FOXG_04696) based on ProTSAV score. **(B)** The ProTSAV score for the template protein 1,3,6,8-tetrahydroxynaphthalene reductase (T4HNR) complexed with NADPH and pyroquilon (1JA9). The ProTSAV evaluated the predicted model structures, based on some popular online servers and standalone tools, and furnishes with a single quality score in case of individual protein structure, along with a graphical representation and ranking in case of multiple protein structure assessment. In our results, the ProTSAV score was found close to 1JA9 which predicts the model has reasonable stability and accuracy in terms of qualitative and quantitative parameters.

The selected model was verified for their stereochemical quality assessment. Furthermore, in each case of qualitative assessment, a comparative study was done with experimentally solved crystal structures, to check the quality, reliability, accuracy, stability and compatibility of the computationally predicted protein. The Ramachandran plot obtained through RAMPAGE server revealed that the predicted model FOXG_04696 had 99.3% residues [97.7% (favored) + 1.6% (allowed)] lying in favored region [compared to the experimentally solved and X-ray resolved template protein structure (3IS3) where we found 98.0% (favored) and 1.6% (allowed) residues against the expected values 98.0% (favored) and 2.0% (allowed) regions]. The other sequential homolog 1JA9 had similar results like 3IS3 98.0% (favored) and 1.6% (allowed). The PROCHECK module of the PDBSum server, further justified the stereochemical goodness of the predicted model, with 94.2% residues accommodating in the most favored regions (A, B, and L) and only 4.9% residues occupied in the additionally allowed regions (a, b, l, and p) with the G factor value 0.12 (Supplementary Figure S5). This confirms the predicted model quality had good stereochemical quality and was close to the template structure. The ProSA results in finding the potential error in the predicted model revealed the *Z* score value −8.11 (Supplementary Figure S6) against the template (3IS3) score value −8.97. The *Z* score of other template (1JA9) was found to be −9.67. The ProSA evaluates the qualitative values of the modeled structures based on atomic coordinates. The energy plots represent the potential problems spotted in protein structures. The *Z* score revealed the protein structures could be correlated well with crystal structures of similar lengths, where the positive value corresponds to problematic or erroneous parts of the input structure. ProQ is a neural network-based predictor based on a number of structural features predicts the quality of protein model. The ProQ result showed LG score of 5.677 which represents that the structure is of very good quality. The ERRAT score for the modeled structure was found to be 90.40% against the template (99.20%) (Supplementary Figure S7). The Verify 3D evaluated that the predicted protein has 91.30% residues had an average 3D-1D score ≥0.2 (Supplementary Figure S8 and Supplementary Table S1). The quality assessment at various interfaces has resulted in a combined ProTSAV score value which revealed that the predicted protein was stable and had RMSD values in the range of a good model (at green–yellow interface) (Figure 6). The VADAR statistics for quantitative evaluation of the predicted model revealed that the model structurally composed of helical (50%) and coil (31%) with interspersed beta sheets (18%) with extensive H bonding groups [donor and acceptor; with the observed value of 83% against the expected 75% score values, and mean H = bond energy −1.7; sd = 1.0 (expected −2.0 sd = 0.8)]. We superimposed the full length predicted protein FOXG_04696 (258 residues) over both the template 3IS3 (260 residues) and the 1JA9 (259 residues) to perform structural alignment, using AuStrAlis server. The RMSD deviations on superposition along the protein carbon backbone were 0.49Å (3IS3) and 1.51Å (1JA9) with the FOXG_04696. This further confirms the results of the qualitative assessment, and structural conservation of SDRs proteins among the closely related group and therefore, their crucial role in the fungal biosystem. These results indicated that the two proteins had a similar structural assignment and topological orientation (functional domain and folds) that predicts their indispensable role. The final predicted models were submitted to an online repository, protein modeling database (PMDB) under the name SDRs (T4HNR: organism name: *Fusarium oxysporum* f.sp. *lycopersici*) and were provided with having accession number PM0081606.

### Active Site Prediction

The putative ligand binding sites (both major and minor) for the predicted protein were identified through Meta-pocket 2.0 server. The conserved domain databases (CDD) server prediction revealed the conservation of the catalytic tetrad (NSYK) ASN^115^, SER^141^, TYR^154^, and LYS^158^ in the FOXG_04696 which was found to be conserved in 1JA9, and were replaced with ASN^138^, SER^164^, TYR^178^, and LYS^182^ with the presence of canonical glycine-rich NADP-binding sites (Supplementary Figure S9 and Supplementary Table S2). By contrast, the C-terminal residues providing specificity for substrate binding (NADP) had conserved active site residues (GLY^13^, ARG^16^, GLY^17^, ILE^18^, ARG^36^, TYR^37^, VAL^38^, SER^39^, SER^40^, ALA^63^, ASP^64^, VAL^65^, ASN^91^, SER^92^, GLY^93^, VAL^114^, ILE^139^, SER^140^, SER^141^, TYR^154^, LYS^158^, PRO^184^, THR^16^, ASP^187^, and MET^188^) compared to the active site residues for template (1JA9) (Supplementary Figure S10). The other substrate binding site in the FOXG_04696 showed the extensive conservation of serine, isoleucine and tyrosine residues SER^141^, ILE^142^, TYR^154^, and TYR^196^ (represented as SER^164^, ILE^165^, TYR^178^, and TYR^223^ in 1JA9) (Supplementary Figure S11 and Table 1). However, at some positions in the FOXG_04696, even the glycine residues were found to be extensively conserved, which reflects their crucial role in NADP binding including GLY^13^, GLY^17^, and GLY^93^ (represented by GLY^36^, GLY^40^, GLY^116^, GLY^209^, and GLY^210^ in 1JA9) which might play an indispensable role and imparts specificity to FOXG_04696.

**Table 1:** Comparative evaluation of the active sites and other binding site residues for T4HNR *Magnaporthe grisea* (1JA9) and the predicted protein FOXG_04696.

T4HNR complexed with NADP(H) and pyroquilon (1JA9) active site residues (X-ray diffraction)	T4HNR *Magnaporthe oryzae* (1JA9) predicted active site (NCBI-CDD results)	FOXG_04696 active site residues (NCBI-CDD results)	Common residues (FOXG_04696 and 1JA9)
GLY^36^ ARG^39^ GLY^40^ ILE^41^ GLY^61^ SER^62^ SER^63^ ALA^86^ ASP^87^ ILE^88^ ASN^114^ SER^115^ GLY^116^ LEU^137^ THR^162^ SER^163^, SER^164^ TYR^178^ LYS^182^ PRO^208^ GLY^209^ GLY^210^ VAL^211^ THR^213^ ASP^214^ MET^215^ PHE^216^ SER^220^ TYR^223^ ILE^282^	**Catalytic tetrad** ASN^138^ SER^164^ TYR^178^ LYS^182^ **NADP-binding residues (substrate)** GLY^36^ ARG^39^ GLY^40^ ILE^41^ ASN^59^ TYR^60^ GLY^61^ SER^62^ SER^63^ ALA^86^ ASP^87^ ILE^88^ ASN^114^ SER^115^ GLY^116^ LEU^137^ THR^162^ SER^163^ SER^164^ LYS^182^ PRO^208^ GLY^209^ GLY^210^ VAL^211^ THR^213^ ASP^214^ MET^215^ **Chemical (fungicide) binding residues** SER^164^ ILE^165^ TYR^178^ GLY^210^ MET^215^ PHE^216^ SER^220^ TYR^223^	**Catalytic tetrad** ASN^115^ SER^141^ TYR^154^ LYS^158^ **NADP-binding residues (substrate)** GLY^13^ ARG^16^ GLY^17^ ILE^18^ VAL^38^ SER^39^ SER^40^ ALA^63^ ASP^64^ VAL^65^ ASN^91^ SER^92^ GLY^93^ GLU^95^ VAL^114^ ILE^139^ SER^140^ SER^141^ TYR^154^ LYS^158^ PRO^184^ LYS^185^ THR^186^ ASP^187^ MET^188^ TYR^189^ ALA^193^ TYR^196^	GLY^13^ ARG^16^ GLY^17^ ILE^18^ SER^39^ SER^40^ ALA^63^ ASP^64^ ASN^91^ SER^92^ GLY^93^ GLU^95^ SER^140^ SER^141^ TYR^154^ LYS^158^ PRO^184^ THR^186^ ASP^187^ MET^188^ TYR^196^

*The active sites for 1JA9 were retrieved through the X-ray-crystal structure and those for FOXG_04696 were retrieved from the NCBI conserved domain database (CDD) server. The common residues were obtained from structural alignment. The common residues present in both have been shown in a separate column.*

### CATH Results

**FIGURE 7:**
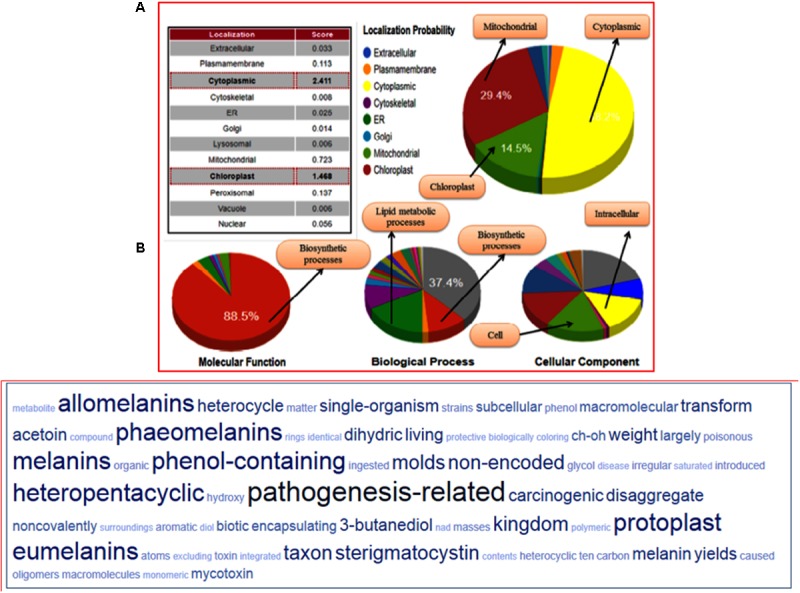
**(A)** Functional annotation of the FOXG_04696 measured in the form of gene ontology enrichment analysis. The three ontological terms used were the molecular function, biological process involved, and cellular location. The sub-cellular localization of the protein was shown as a big pie chart and were retrieved through the Cello predictor. **(B)** The tag cloud diagram showing the keywords that are frequently associated with the FOXG_04696 protein and indicates its probable function in biosynthetic mechanisms and metabolism.

The structural classification through CATH server revealed that the predicted model belongs to (α+β) type (3), (A) three layer (aba) sandwich type architecture (3.40), having Rossmann fold (3.40.50) and bearing to NADP binding Rossmann fold (alpha/beta folding pattern with a central beta-sheet) like domain family protein (30.40.50.720). The functional annotation using Funfam (functional families) revealed the possible biological role of the characterized protein based on three ontological terms that include biological process, molecular function, and cellular component. The first five significant GO terms in biological processes included secondary metabolite biosynthetic process (GO: 0044550), secondary metabolite process (GO: 0019748), pigment biosynthetic process (GO: 0046148), and sterigmatocystin biosynthetic process (GO: 0045461). The significant terms in molecular processes found were versicolorin reductase activity (GO: 0042469), tropinone reductase activity (GO: 0050358), NAD^+^ binding (GO: 0070403), (S, S)-butanediol dehydrogenase activity (GO: 0047512), and alcohol dehydrogenase (NAD) activity (GO: 0004022). The scattered plot diagram was generated through the ReviGO web server was based on non-redundant GO terms with scoring values higher is better. The first five significant terms structured around three ontologies, which discussed biological processes, molecular function and a subcellular component of predicted protein has been shown (Supplementary Figure S12). The subcellular localization and function annotation were further revealed through the CELLO2GO server discussed the queried protein sequence, was found to be associated with biosynthetic and secondary metabolism processes, with having an oxidoreductase activity (88.4%) (Figure 7A). The tag cloud diagram describes the frequent keywords associated with the assigned GO terms, and therefore, represents the functional relevance of the proteins and the other associated processes in which their function have been elucidated (Figure 7B).

### Protein–Fungicide Interaction

**FIGURE 8:**
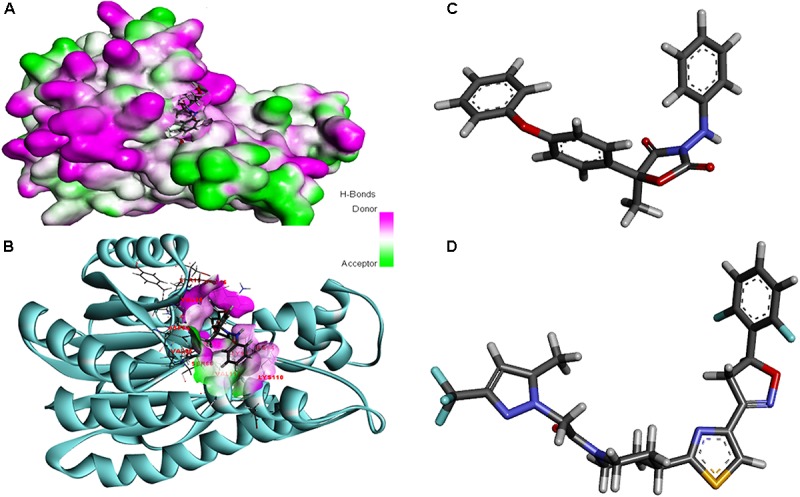
**(A)** The overall 3D surface view of the modeled protein FOXG_04696 represented to display all the possible H-bond donor and acceptor group when complexed with ligand (Famoxadone). **(B)** The interaction of the ligand (Famoxadone) with protein FOXG_04696 with the possible H-bond donor and acceptor groups, near the ligand interacting or binding sites (active sites). **(C)** The 3D representation of the ligand Oxathiapiprolin. **(D)** The 3D structure of the ligand Famoxadone.

The modeled protein FOXG_04696 was docked with all the 37 fungicides to generate their binding mode and dynamic simulations was done to refine the best pose with allowed conformational change in the FOXG-04696 (Rachman et al., 2018). We have evaluated the protein–fungicide interaction through YASARA and Glide-based molecular docking program. It was found that both the tools have discrepancies in results for accurate pose prediction among the various putative docking poses, revealed through scoring functions, which might leads into conclusion that, docking scores are not sufficiently precise to represent the protein ligand binding affinity (Suenaga et al., 2012). MD simulations analysis of the docked complexes discriminated the correct docking poses from decoy poses, as the unstable and incorrectly docked structures during MD simulations results into unstable trajectories that finally lead into disruption of the complex. By contrast, the realistic complexes provide stable behavior (Yunta, 2016). Furthermore, based on obtained MD trajectories, Δ*G*_bind_ was computed by using MM/GBSA calculations. In many studies, it has been demonstrated that binding free energies predicted by MM/GBSA-based rescoring of the docked complexes are in good agreement with experimental binding affinities (Suenaga et al., 2012; Shen et al., 2013). The Oxathiapiprolin had the least Δ*G*_bind_ of –75.50 (±0.54) kcal/mol and XPG docking score of −1.86 kcal/mol with 17 binding site residues (LEU^100^, VAL^103^, ILE^108^, LEU^112^, VAL^116^, TRP^146^, GLY^147^, VAL^148^, PRO^149^, ARG^150^, HIS^151^, ALA^152^, LEU^153^, SER^155^, ALA^156^, SER^157^, and ALA^160^) of the T4HNR were found to involve in van der Waals interactions with Oxathiapiprolin. The Famoxadone had the Δ*G*_bind_ of −66.90 (±0.47) kcal/mol and lower XPG score (than Oxathiapiprolin) of −3.30 kcal/mol, it displayed two hydrogen bonds with key binding site residues TYR^154^ and THR^186^ and 27 residues were found to be involved in making van der Waals interactions GLY^13^, SER^15^, ARG^16^, GLY^17^, ILE^18^, GLY^19^, TYR^37^, VAL^38^, ASN^91^, SER^92^, GLY^93^, ILE^94^, GLU^95^, ILE^139^, SER^140^, SER^141^, ILE^142^, SER^143^, TYR^154^, LYS^158^, PRO^184^, LYS^185^, THR^186^, ASP^187^, MET^188^, TYR^189^, ALA^192^, ALA^193^, and TYR^196^) within 4Å binding site region of Famoxadone with T4HNR. The 3D surface view of the docked Famoxadone–FOXG_04696 complex has been shown to represent the putative H bond acceptor and donor group (Figure 8A). The functional H bond acceptor and donor group from protein major binding sites of proteins have been shown in Figure 8B. The 3D structure of two effective ligands (fungicides) has been shown in Figures 8C,D.

**FIGURE 9:**
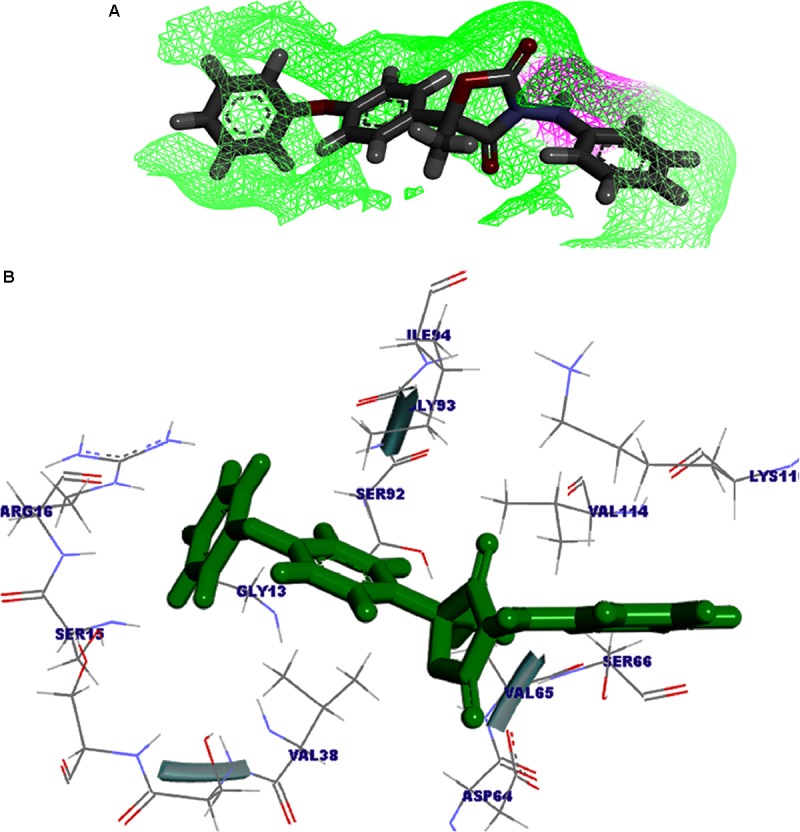
**(A)** The ligand Famoxadone represented through the wire mesh diagram to show the probable H-bond donor or acceptor groups that could have crucial role in protein–fungicide interaction. **(B)** 3D representation of the ligand molecule when docked with the FOXG_04696 showing the crucial residues of protein that have important contribution in binding with the ligand donor or acceptor group.

The protein–fungicide docking was further analyzed through the YASARA, an auto dock based tool for molecular docking and virtual screening to calculate the docking score (kcal/mol) and dissociation constant (*K*_d_) μM. The maximum YASARA score was found to be associated with the Oxathiapiprolin (7.81 kcal/mol) with least dissociation constant *K*_d_ value 1.86 (μM) followed by the Famoxadone (7.65 kcal/mol; *K*_d_ value 2.43 μM). The protein–ligand docking through the YASARA showed the efficient, stronger, and stable binding with positive YASARA score^∗^ (YASARA scoring^∗^, where positive energy means stronger binding and negative energy means no binding) (Chen et al., 2015) with Oxathiapiprolin followed by Famoxadone. The putative H bond acceptor and donor group in ligand Famoxadone were shown through a receptor mesh diagram (Figure 9A). The 3D diagram of the Famoxadone that interacted with crucial residues from the major binding site has been shown (Figure 9B).

The YASARA based soring, dissociation constant, and contact receptor residues involved in binding with the FOXG_04696 have been shown in Supplementary Table S3. Since the fungicide pyroquilon is an efficient fungicide used against the rice blast pathogen (*M. grisea* PDB ID: 1JA9), and targets the residues, forming active sites of the T4HNR (SDR) protein. We have investigated the X-Ray determined crystal structure of the T4HNR (1JA9) complexed with fungicide pyroquilon to find out the residues that were involved in binding with T4HNR in an accurate and flexible docking poses (Supplementary Figure S13). The investigation revealed that pyroquilon docked with maximum residues that constituted the major binding sites (active site). In this way, one could predict that the fungicides that target the active site residues of T4HNR protein with maximum interacting residues (more accurate docking pose) and better protein ligand contacts (flexible docking) could have better binding efficiency, and therefore, would be useful for disrupting the functional mechanism of T4HNR. In our results, we have evaluated the comparative docking efficiency (pyroquilon as control) to investigate the binding affinity measured in the form of YASARA-based docking score, and dissociation constant of the docked complexes. The residues involved in making feasible and accurate docking of T4HNR with pyroquilon were GLU118, SER^164^, ILE^165^, ALA^166^, TYR^178^, PRO^208^, GLY^209^, GLY^210^, MET^215^, PHE^216^, ASN^219^, SER^220^, TYR^223^, LEU^240^, and ILE^282^. The computational screening and docking studies of 37 fungicides with the FOXG_04696 revealed that Oxathiapiprolin followed by the Famoxadone binds with maximum YASARA score and least dissociation constant (*K*_d_).

The molecular docking and virtual screening through Glide XP ranked ligands based on an accurate pose prediction(the ligand ability to bind for a specific receptor conformation) for each-protein—fungicide complex in order to separate those ligands that don’t bind, in a ranked list. Furthermore, analysis of the YASARA results for the two top scored docked protein–fungicide complexes (Oxathiapiprolin and Famoxadone) revealed that Famoxadone docked with FOXG_04696 in an accurate and flexible docking pose with residues that constituted the major binding site (active site including the catalytic tetrad) GLY^13^, SER^15^, ARG^16^, VAL^38^, SER^39^, SER^40^, ASP^64^, VAL^65^, SER^66^, SER^92^, GLY^93^, ILE^94^, LYS^110^, and VAL^114^. Since, we did not find any stable docking conformation for stable binding of the Oxathiapiprolin at that particular specified docking site (like Famoxadone). The lower ranking of the Oxathiapiprolin (higher XPG score) binding over the Famoxadone (lower XPG score) could be interpreted from the fact that the Oxathiapiprolin was found to docked in an alternative conformation docked with the residues that were either absent from any major or minor binding site, or were present beyond the limit required for an accurate docking pose prediction. The visualization of the Glide XP docked complexes revealed that Oxathiapiprolin bounded with LEU^100^, VAL^103^, ILE^108^, LEU^112^, VAL^116^, TRP^146^, GLY^147^, VAL^148^, PRO^149^, ARG^150^, HIS^151^, ALA^152^, LEU^153^, SER^155^, ALA^156^, SER^157^, and ALA^160^ rather than the specified docking sites. The molecular complexes formed after protein–fungicides interaction for different fungicides has been visualized through the visualization tool of Discovery studio 3.0 and have been represented (Supplementary Figure S14).

### MD Simulations

**FIGURE 10:**
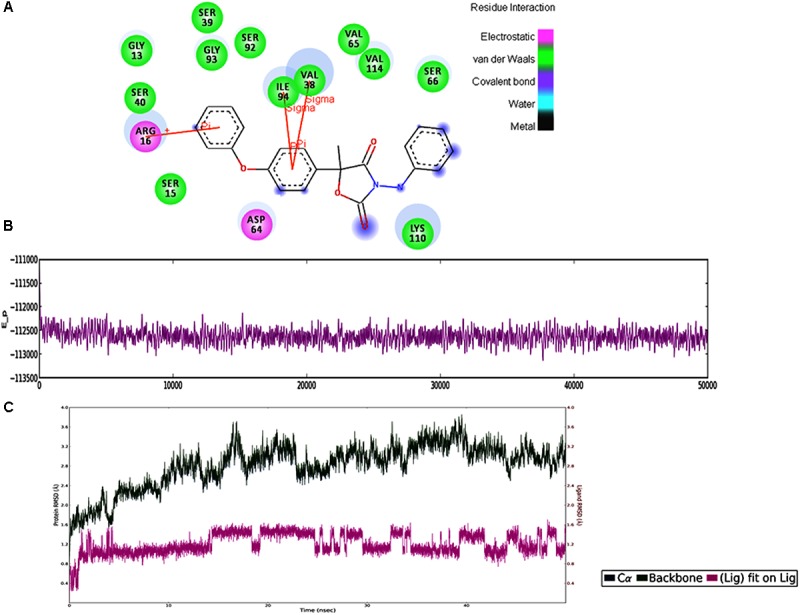
**(A)** The 2D representation for the docked complex of the FOXG_04696. The figure showed the putative residues involved in interaction with Famoxadone. The different colors have been used for showing the different types of molecular interactions involved. **(B)** MD simulations trajectories for the FOXG_04696–Famoxadone complex showing the average potential energies of the docked complexes during the 50-ns MD simulations. **(C)** Plot of the root mean square deviation (RMSD) of Cα of T4HNR (protein) and the FOXG_04696–Famoxadone (complex). RMSDs were calculated using the initial structures as templates.

**FIGURE 11:**
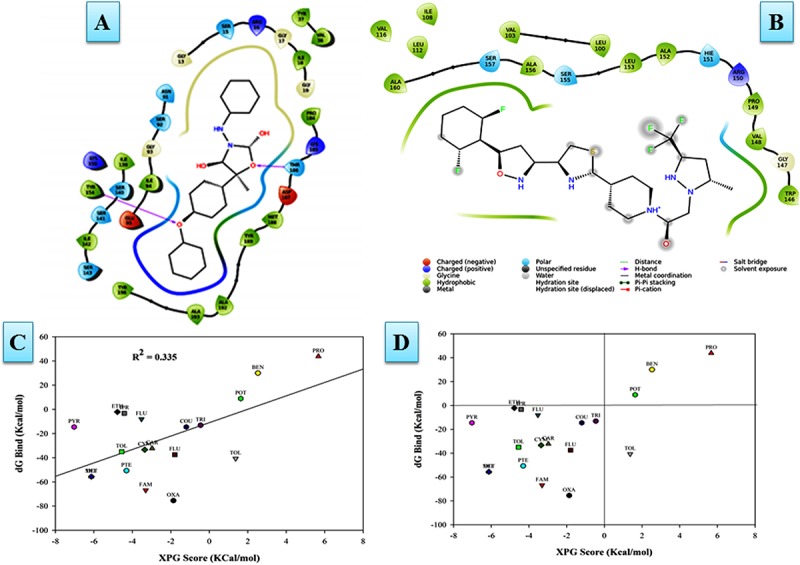
**(A)** The Oxathiapiprolin–FOXG_04696 interaction results during 50-ns MD simulations. **(B)** Interaction of the Famoxadone with FOXG_04696 showing the residues involved in protein–fungicide docking with different type of molecular interactions. **(C)** The correlation plot showing the values of correlation coefficient *R*^2^ = 0.335 based on binding affinities (kcal/mol) and MM/GBSA (Δ*G*) binding free energy calculations showing the strong correlation between the binding free energies Δ*G*_bind_ and binding affinities for ranking/scoring of the fungicides in the docked complexes. The three later symbols with different colors have been used for representing the ligands. **(D)** The scatter plot displaying docking (XPG) and binding free energy MM/GBSA (ΔG_bind)_ scores represented in the quadrant view form for all the 19 protein–fungicide docked complexes.

In MD simulations analysis, the FOXG_04696–Oxathiapiprolin complex had an average potential energy of −113166.16 kcal/mol which disclosed the steadiness of the complex. The average RMSD for the FOXG_04696 backbone and the Oxathiapiprolin were 2.49 and 2.42Å, respectively (Supplementary Figure S15A). The average RMSF for backbone and side chain for the FOXG_04696 accommodating with the Oxathiapiprolin were 1.54 and 1.70Å, respectively (Supplementary Figure S15B). Oxathiapiprolin–FOXG_04696 complex exhibited five hydrogen bonds with water (SER^143^, ALA^144^, VAL^145^, GLY^147^, and SER^155^), three water mediated hydrogen bonds (VAL^148^, ARG^150^, and LYS^166^), four hydrophobic and water-mediated hydrogen bonds (TRP^146^, ALA^152^, ALA^156^, and ALA^159^) with seven hydrophobic interactions (LEU^100^, VAL^103^, ILE^108^, LEU^112^, PRO^149^, LEU^153^, and ALA^160^) with the key binding site residues to form a stable complex (Supplementary Figure S15C). We have shown the protein–ligand interaction 2D diagram as visualized through the Discovery Studio 3.0 (Figure 10A). By contrast, the FOXG_04696–Famoxadone complex has an average potential energy of −112628.96 kcal/mol disclosed the steadiness of this complex (Figure 10B). The average RMSD for the T4HNR backbone and the Famoxadone were found to be 2.83 and 1.20Å, respectively (Supplementary Figure S10C). The average RMSF for the backbone and side chain of the T4HNR to accommodate the Famoxadone were reported 1.30 and 1.86Å, respectively (Supplementary Figure S15D). The Famoxadone exhibited seven hydrogen bonds (ASN^91^, SER^92^, GLY^93^, GLU^95^, SER^141^, THR^186^ and ASP^187^) and 15 hydrophobic interactions (GLY^13^, ARG^16^, ILE^18^, VAl^38^, VAL^65^, ILE^94^, ILE^139^, ILE^142^, TRP^146^, LYS^158^, PRO^184^, MET^188^, TYR^189^, ALA^192^, and TYR^196^) with the key binding site residues in forming a stable complex (Supplementary Figure S15E). Both the complexes were relatively stable with the lesser average potential energy but Famoxadone displayed more interactions with FOXG_04696 compared to Oxathiapiprolin in 50 ns MD simulations, with lesser RMSD and RMSF values and best protein ligand contacts among all the docked 37 fungicides for the specified docking site (active site or major binding site). The 3D representations for the protein–fungicide interaction for both Oxathiapiprolin (Figure 11A) and Famoxadone have been shown (Figure 11B). The correlation plot showing the values of correlation coefficient *R*^2^ = 0.335 based on binding affinities (kcal/mol) and MM/GBSA binding free energy (Δ*G*_bind_) calculations showing the strong correlation between the predicted binding free energies and ranking affinities/scoring of the fungicides for the docked complexes (Figures 11C,D).

We have compared our docking results, both from the Glide XP docking and the YASARA protein–ligand docking tool. Furthermore, the docked complexes were rescored through MM/GBSA free energy binding calculations to validate the docking based results for ranking the binding affinities of docked ligands (fungicides). The ultimate goal for MM/GBSA calculations was to estimate the enthalpy change on ligand binding, through comparison of an average enthalpy change for the bound and unbound states. The MM/GBSA results re-ranked the docked complexes in terms of their accurate pose prediction and efficacy for binding affinities (Table 2). We did not find any significant docking pose for the Oxathiapiprolin–FOXG_04696 complex, particularly on the specified docking site (active site including catalytic tetrad) of (FOXG_04696) even at minimized grid space. However, MD simulations of the FOXG_04696–fungicide docked complexes revealed the stable binding of the Famoxadone over the Oxathiapiprolin with all crucial residues occupying interactions with ligand in MD simulations. The 2D diagram of the protein–fungicide complexes, when visualized through the Discovery Studio 3.0 tool, we found some interesting results. The YASARA-based docking with Famoxadone was found comparable to the MD simulations results, as the residues involved in the protein–ligand contact were found to be similar, and were found to be involved/constitute the major binding (active sites) of the FOXG_04696. The YASARA based docking score and dissociation constant obtained has been plotted (Supplementary Figure S16). By contrast, the Oxathiapiprolin–protein complexes, when analyzed were found to have maximum interacting residues for sites that constitute the minor binding sites or second probable binding site (metapocket results; Supplementary Table S1). The MD simulations and the YASARA based docking for the Famoxadone-protein was found to have residues from a major binding site that include GLY^13^ SER^15^, ARG^16^, VAL^38^, SER^39^, SER^40^, ASP^64^, VAL^65^, SER^66^, SER^92^, GLY^93^, ILE^94^, LYS^110^, and VAL^114^. By contrast, the YASARA based Oxathiapiprolin–protein complex was found to have residues like GLY^13^, ARG^16^, VAL^38^, ASP^64^, VAL^65^, SER^66^, LYS^67^, SER^92^, GLY^93^, ILE^94^, ASP^109^, LYS^110^, LEU^112^, GLY^113^, and VAL^114^, whereas the MD simulations analysis covered the residues not lying in major binding site (meta-pocket results) or located at other binding cavities rather than the residues that were involved in the main binding sites (Supplementary Table S4).

**Table 2:** Comparative evaluation of protein-ligand (fungicide) docking interactions from YASARA programme and XP Glide score (docking score) values.

S. No.	Fungicide	YASARA score	Dissociation constant (*K*_d_) (μM)	XPG score (kcal/mol)	MM/GBSA (Δ*G*_bind_) (kcal/mol)
1.	Oxathiapiprolin	7.81	1.86	−1.89 ± 0.32	−75.50 ± 0.54
2.	Famoxadone	7.65	2.43	−3.30 ± 0.28	−66.90 ± 0.47
3.	Metiram	4.10	976.17	−6.13 ± 0.25	−55.63 ± 0.38
4.	Dithane	4.10	976.17	−6.13 ± 0.22	−55.63 ± 0.42
5.	Pterostilbin	6.29	24.17	−4.30 ± 0.20	−50.69 ± 0.56
6.	Tolclofos-methyl	4.91	249.18	1.38 ± 0.10	−40.70 ± 0.47
7.	Fluberidazole	6.63	13.64	−1.79 ± 0.33	−37.50 ± 0.52
8.	Tolprocarb	6.64	13.50	−4.55 ± 0.64	−35.02 ± 0.64
9.	Cymoxanil	5.70	57.90	−3.35 ± 0.24	−33.39 ± 0.76
10.	Carbendazim	5.78	57.09	−2.98 ± 0.38	−32.28 ± 0.49
11.	Coumarin	6.13	32.05	−1.19 ± 0.19	−14.58 ± 0.36
12.	Pyraclostrobin	7.05	6.69	−7.02 ± 0.10	−14.57 ± 0.39
13.	Triazoquinoline	6.43	19.25	−0.45 ± 0.30	−13.19 ± 0.33
14.	Fludioxonil	5.98	40.87	−3.53 ± 0.24	−8.07 ± 0.13
15.	Iprodione	5.98	41.08	−4.42 ± 0.34	−3.30 ± 0.31
16.	Ethyl phosphonate	5.05	197.07	−4.78 ± 0.25	−2.14 ± 0.51
17.	Prothioconazole	5.53	87.65	1.64 ± 0.11	8.94 ± 0.43
18.	Benomyl	5.58	80.15	2.53 ± 0.39	29.98 ± 0.74
19.	Prochloraz	5.43	103.25	5.68 ± 0.36	43.86 ± 0.80

*The YASARA based dissociation constant (Kd) have been given in a separate column. The docked complexes were further rescored for binding free energy assessment using the MM/GBSA method. The MM/GBSA based binding free energies have been arranged in the increasing order, reflects the decreasing order of stability and steadiness of the complexes. The Glide based interaction of the protein-fungicide docking complexes and binding free energy assessment through MM/GBSA were set up with three replication and data were analyzed by Mean (± SE) was calculated from three replicates for each of the docked complexes. We have shown the docking score values only for the significant docked protein-ligand complexes (19 fungicides; as others were found unable to dock at the intended site through Glide XP dock and also had positive free energies as calculated through MM/GBSA approach.*

### Protein–Protein Interaction Network

**FIGURE 12:**
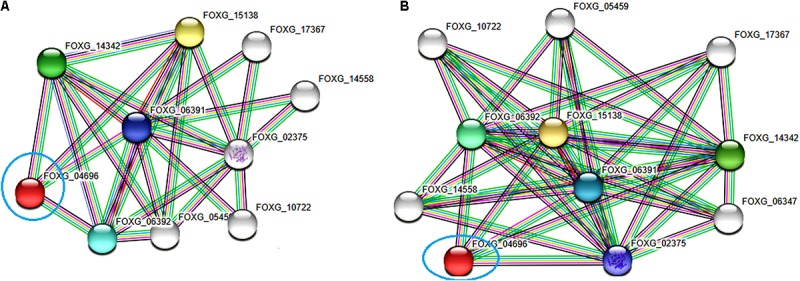
The protein–protein interaction associative network for the FOXG_04696 through STRING server. The active interaction sources were set based on the seven parameters including experiments, co-expression, gene fusion, co-occurrence, databases, text mining, and neighborhood. **(A)** The interactions analyzed at the highest confidence level (0.90) with maximum five interacting partners from both shells of interactors. **(B)** Interaction at high confidence level (0.70). The color nodes describe query proteins and the first shell of interactors, whereas white nodes are the second shell of interactors. The large node size represents characterized proteins and smaller nodes for uncharacterized proteins.

The functional interactive network formed by the FOXG_04696 protein at the highest confidence level (0.90) has been shown in Figure 12A. The predicted protein was shown to have an interaction with the fatty acid synthase subunit beta dehydratase (FOXG_06392) and the fatty acid synthase subunit alpha dehydratase (FOXG_06391). However, at high confidence level (0.70), we found the interaction of our predicted protein FOXG_04696 with acetyl-CoA carboxylase (FOXG_02375; interacting score 0.847). The interaction network of FOXG_04696 at high confidence level has been shown in Figure 12B. The interactive associative protein network formed by various interacting partners, with their interacting score annotation identities and accession identities values have been shown (Supplementary Table S5).

### *In vitro* Assessment of Fungicides

**FIGURE 13:**
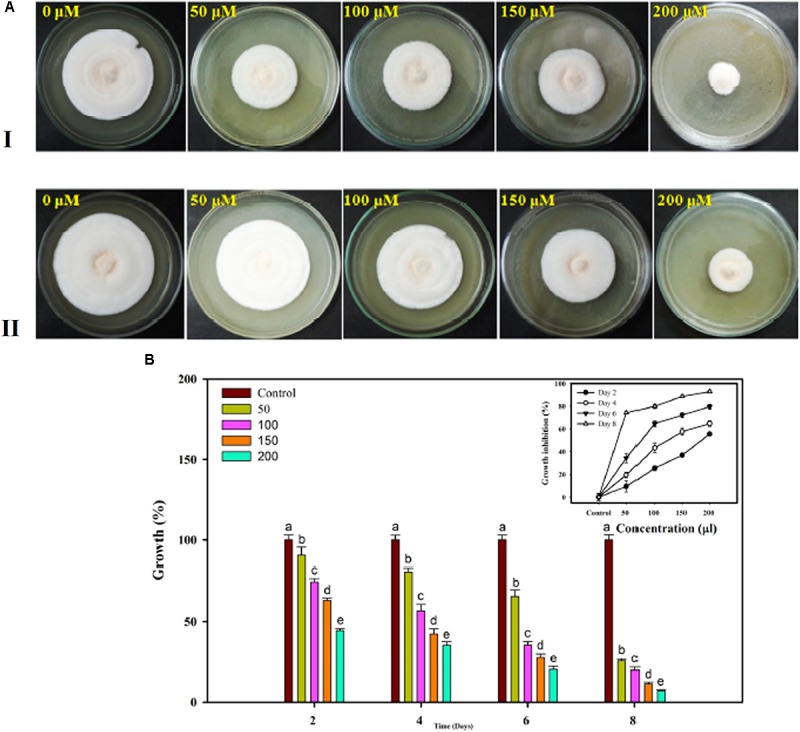
*In vitro* assessment of the fungicide on growth response of FOL pathogen. Four different concentrations were selected 50, 100, 150, and 200 μL along with control at 4 days interval **(A-I)** and at 8 days interval **(A-II)**. **(B)** The growth was recorded at even day’s interval (2, 4, 6, and 8) and the percentage inhibition was calculated using statistical tools.

The Famoxadone solution used for *in vitro* assessment against the FOL pathogen showed growth inhibition at each and every increasing concentration of fungicides. With increasing concentrations of fungicides, the growth rates were correspondingly retarded and sporulation was reduced. The maximum growth inhibition was recorded on eighth day post inoculation. The percentage inhibitions measured in the form of radial growth were subjected to statistical analysis. The percent inhibitions recorded on 4th day were 90.53, 74.42, 63.04, and 44.36 at 50, 100, 150, and 200 μL concentrations, respectively. By contrast, the percent inhibitions recorded on 8th day post inoculation were 25.73, 19.99, 11.22, and 7.04 at 50, 100, 150, and 200 μL concentrations, respectively. The growth inhibition recorded on the 4th and 8th days at different concentrations of fungicides has been shown in Figures 13A-I,II, respectively. The statistical data for growth measured at different concentrations and on even days have been shown in bar diagram (Figure 13B).

### *In silico* Toxicity Assessment

The Famoxadone was checked and evaluated for toxicity assessment for its safe environmental disposition. The FAF-drugs 4.0 tool performed the computational prediction of some ADME-Tox properties (adsorption, distribution, metabolism, excretion, and toxicity) for Famoxadone and it was found that the drug is non-carcinogenic and acceptable (Yadav et al., 2017). Furthermore, the ligand (Famoxadone) was found to follow Lipinski Rule of five for drug likeness with molecular mass 374.000000 (<500 Da), hydrogen bond donor 1, hydrogen bound acceptor 6, with Log *P* score values 4.699, and molar refractivity 103.70. The admetSAR results predicted that the selected ligand (Famoxadone) was found to be non-carcinogen (0.7751), non-AMES toxic (0.5395), and non-inhibitor (0.8941), with weak hERG (the human Ether-à-go-go-Related Gene) inhibitor (0.9732) and with non-required carcinogenetic (0.4799).

## Discussion

Vascular wilt caused by *F. oxysporum* f.sp. *lycopersici* (FOL) is very destructive and widespread plant disease that causes enormous economic losses. The wilt pathogen directly penetrates roots and colonizes the vascular tissue (Inoue et al., 2002). One of the most important characteristics of Fusarium wilt disease is the discoloration of vascular tissues, which is due to the brownish-black melanoid pigment. Melanin biosynthesis, therefore, is a good target for designing the antifungal agents. The biosynthesis of fungal melanin is derived from a pentaketide intermediate which cyclized into 1,3,6,8-tetrahydroxynaphthalene. The final step of the reaction is accomplished by series of reductions and dehydrations and forms 1,8-dihydroxynaphthalene (DHN) through the formation of intermediates including (+) scytalone, 2,3,8-trihydroxynaphthalene (T, H, and N). DHN may be then oxidized and polymerized to form melanin (Bell and Wheeler, 1986; Feng et al., 2001). The 1,3,6,8-tetrahydroxynaphthalene/1,3,8-trihydroxynaphthalene reductase gene has been isolated from *M. grisea* (Vidal-Cros et al., 1994). The melanin biosynthetic pathway was recently demonstrated in other Ascomycetous fungi based on sequence similarity, percent identity to the T4HNR protein (encoded by *teh* gene). Engh et al. (2007) reported the DHN-based melanin pathway in the *Sordaria macrospora*, an Ascomycetous fungal model system, which accumulates the melanin during its sexual development. It was found that the T4HNR protein showed sequence similarity and homology with *Aspergillus fumigatus* (taxid: 746128) (51.8% identity), *Cochliobolus heterostrophus* (taxid: 5016) (79.1% identity), *M. grisea* (taxid: 148305) (51.6% identity), and *Neurospora crassa* (taxid: 5141) (96.3% identity). By contrast, the polyketide synthase (encoded by pks gene) (DHN melanin enzyme) had sequence similarity with *A. fumigatus* (42.2% identity), *C. heterostrophus* (46.0% identity), *Colletotrichum lagenarium* (66.5% identity), *M. grisea* (69.6% identity), and *N. crassa* (85.6% identity) (Engh et al., 2007). The orthologs for the genes encoding for enzymes polyketide synthase (*pks*), a tetrahydroxynaphthalene reductase (*teh*), a scytalone dehydratase (*sdh*), and a trihydroxynaphthalene reductase (*tir)* were used from other Ascomycetous fungi (mentioned above) to retrieve the sequences of the above genes and further for their experimental demonstration in *S. macrospora* (Engh et al., 2007). Interestingly, the polypeptide products obtained after comparative sequence analysis had significant homology to DHN-melanin pathway enzymes of other filamentous ascomycetes, and confirm the role of DHN melanin in *S. macrospora*. (Engh et al., 2007). By contrast, the homology search of the FOXG_04696 (XP_018239507.1) with *S. macrospora* (TER) and *M. grisea* (TER), the protein Blast-p results revealed the 99% sequence query coverages and 51% sequence identity with *S. macrospora* (XP_003345723.1), 93% query coverages and 49% sequence identity with *M. grisea* (TER) (PDB ID 1JA9), and only 91% query coverages and 46% sequence identities with *M. grisea* (TIR). Based on such *in silico*-based comparative studies, one could predict the existence of DHN melanin pathways in other filamentous fungi (Engh et al., 2007). However, the reduction reactions in the fungal DHN melanin pathway can be performed by only one hydroxynaphthalene reductase, whereas other ascomycetes (*M. grisea and S. macrospora*) utilize two reductases the 1,3,8-THN reductase (3HNR) and the 1,3,6,8-THN reductase (T4HNR). In other cases, scytalone dehydratase was considered to activate both dehydration steps, of the scytalone and vermelone (Bell and Wheeler, 1986). Based on Blast-p annotation, we have found only one hydroxynaphthalene reductases (T4HNR) in the FOL pathogen, and more identical in sequential homology to the *S. macrospora* rather than the *M. grisea*. The Blast-p search revealed the sequential similarity and homology with our target protein (the FOXG_04696) with 49% identity and 93% (query coverages) (FOXG 04696: XP_018239507.1). By contrast, the Blast-p results with the *M. grisea* 1,3,8-trihydroxynaphthalene reductases (TIR) (PDB ID: 1G0O) revealed the lesser sequence similarity with the FOXG_04696 (46% identity and 91% query coverages) which reflected, the identity of our target protein as the T4HNR and were found to be more closer to the *M. grisea* T4HNR (1JA9). Moreover, the scytalone dehydratase (EC: 4.2.1.94) protein (KEGG ID: FOXG_13320; Uniprot ID: A0A0D2YAJ4; NCBI ID: XP_018252510.1) and the mRNA (XM_018393275.1) have been well characterized in the FOL pathogen.

The DHN pathway for melanin biosynthesis is reported in many other fungi including *Ophiostoma floccosum.* It was demonstrated that the hydroxynaphthalene reductases (HNR) of the fungus *O. floccosum*, shared the functional homology with other fungal HNR (Eagen et al., 2001). For instance, the HNR reductases deficient *buf* mutant of the rice blast fungus *M. grisea* when provided with the functional HNR reductases *of O. floccusm*, the complemented *M. grisea buf* mutants produced a black pigment like a wild-type strain, and the mutants were found to restore the pigment biosynthesis, which predicted that the existence of functional homology exists in between the fungal genera for the melanin biosynthetic mechanism (Eagen et al., 2001).

It was reported that the polyketide synthases involved in fungal DHN melanin biosynthetic pathways belong to the group of iterative type I polyketide synthases similar to fatty acid synthases (Hopwood and Sherman, 1990) and the PKS reported for *S. macrospora* was predicted to contain a β-ketoacyl synthase, two acyl carrier protein domains, thioesterase, an acetyl transferase, and two acyl carrier protein domains. Furthermore, the comparative analysis of non-ribosomal peptide synthetases (NRPSs) and polyketide synthases (PKSs) of 12 different species belonging to *Fusarium* genera revealed the 52 NRPSs and 52 PKSs orthology group (Hansen et al., 2015). The study revealed the conservation of eight NRPSs and (NRPS2–4, 6, 10–13) and two PKSs (PKS3 and PKS7) (Hansen et al., 2015). However, existence of the DHN based melanin in the FOL is rather controversial as it was reported that the PKS encoding gene for DHN melanin biosynthesis is not present in bikaverin producing *Fusarium* genera including *F. verticilloides*, *F. oxysporum*, and *F. fujikuroi* (Kroken et al., 2003). By contrast, Amany and Ellil (2005) characterized the brown colored melanin, in the FOL pathogen, and also evaluated sensitivity of the FOL pathogen against the Tricyclazole and Chlobenthiazone (melanin biosynthesis inhibitor). In the last few years, several melanin biosynthetic inhibitors have been designed to target various phytopathogenic fungi. Furthermore, FOXG_04696 have been shown to have alcohol dehydrogenase (NAD) (GO: 0004022) and NADH binding activity (GO: 0070404). Corrales et al. (2011) reported an alcohol dehydrogenase gene (SDR), *adh*1, has dual fermentative and oxidative functions, and is involved in the fungal (FOL) virulence in tomato plants. In this context, the functional relevance of the FOXG_04696 could be predicted from conserved functional motif and domains, measured in terms of gene ontology, and/or shared domain–domain interaction. Since, the protein structure is 3–10 times more conserved than its sequence (Illergård et al., 2009), and the shared protein domains might be useful for structural and functional annotation of genes or their encoded products. This could be possibly employed to evaluate the molecular functions and biological processes of interacting proteins or domains. The functional annotation as revealed through CATH server revealed that the FOXG_04696 belong to SDR family and might have possible role in versicolorin reductase activity (GO: 0042469), tetrahydroxynaphthalene reductase activity (GO: 0047039), (S,S)-butanediol dehydrogenase activity (GO: 0047512), and tropinone reductase activity (GO: 0050358). The significant biological process measured in terms of gene ontology was melanin biosynthetic process (GO: 0042438), secondary metabolite (bikaverin, fumonisins, fusaric acid, and fusarins) biosynthetic processes (GO: 0044550), pigment biosynthetic process (GO: 0046148), sterigmatocystin biosynthetic process (GO: 0045461), butanediol metabolic process (GO: 0034077), and acetoin metabolic process (GO: 0045149). The structure of the FOXG_04696 was predicted based on comparative modeling. It was reported that up to date, comparative modeling is the most successful and accurate method as evolutionarily related proteins usually share a similar structure (sequence identity >30%) (Errami et al., 2003; Choong et al., 2011) and structural dynamics is the cornerstone of the protein function and its regulation (Berezovsky et al., 2017).

The functional conservation of protein homology was also evaluated based on the protein interaction networks. Since, protein–protein interaction studies are mediated by a limited set of domain–domain interactions (Itzhaki et al., 2006; Reimand et al., 2012), and protein domains represent the structural, functional, and evolutionary unit of proteins (Vogel et al., 2004; Jin et al., 2009). The STRING based results for finding the T4HNR interacting partner in *S. macrospora* revealed that at confidence level from high to highest the T4HNR (*teh*) interacted with fatty acid synthase alpha subunit reductase (XP_003349949.1), fatty acid synthase beta subunit dehydratase (XP_003349948.1), and 3-oxoacyl (acyl carrier protein) synthase (XP_003351602.1). Moreover, the STRING based results for our characterized and predicted model the FOXG_04696, revealed the same interacting partner at highest confidence level values such as fatty acid synthase subunit alpha-reductase (FOXG_06391), fatty acid synthase subunit beta hydratase (FOXG_06392) (enoyl-[acyl carrier protein] reductase (NADH) activity) fatty acid synthase subunit beta hydratase (FOXG_15138), and fatty acid synthase subunit beta-dehydratase (FOXG_14342) indicating the similar functional association with the FOXG_04696 protein.

### *In silico* Characterization and Model Validation

The functional characterization of both template 1JA9 and the predicted model FOXG_04696) through the ScanPROSITE program revealed that the input protein sequences have signature sequences, belonging to SDR family. A broad range of different activities is catalyzed by the enzyme (SDRs) that includes metabolism of organic biomolecules such as carbohydrates, lipids, amino acids, steroids, cofactors, and aromatic compounds and act in redox sensing (Tang and Le, 2014). Sequence analysis revealed that the FOXG_04696 belongs to classical SDRs and has the conserved catalytic tetrad (NSYK) composed of ASN^115^ SER^141^ TYR^154^ and LYS^158^. The three conserved residues including SER^141^, TYR^154^, and LYS^158^ form the structural motif with ASN^115^ through H bonding with other residues. The superimposition of the template protein over the predicted model resulted into the structural resemblances with the minimum RMSD (0.47Å) and the relative RMSD values (0.025Å). The superposition results also aligned the identical residues found in between the T4HNR and the FOXG_04696 protein. The optimized model was found to be suitable based on several qualitative backgrounds including the RAMPAGE, ProSA, ERRAT, PROCHECK (PDB Sum), and Verify-3D. The Ramachandran plot which evaluated that the predicted models were closer to the template (98% residues lying in the favored regions). The ERRAT score values (92%) and Verify-3D results were good enough signifying the consistency of the model prediction and explained that the predicted model was reliable and satisfactory, as it was reported that, for a model having good resolution (approximately 2.5–3.0Å), the ideal score values for Verify-3D should be 80%, and that for the ERRAT around 95% (Colovos and Yeates, 1993). Furthermore, the predicted model was measured in terms of its quality from PROSA score values. The *Z* score value for the predicted structure was −8.07 (against the X-ray resolved template protein having *Z* score value −9.63), which is within the range observed for the native set of proteins of the same size. It was reported that the *Z* score values for any modeled structure lying outside the range of native proteins that were resolved through X-ray and NMR predict the erroneous structure (Wiederstein and Sipp, 2007). This was also confirmed from the ProtSAV score values as all the qualitative parameters measured the predicted model lying in the zone of good resolution (2.5–3.0Å).

In our results, we have evaluated and compared the binding efficacy of commercial fungicides that could be used against the FOL pathogen to control the Vascular wilt disease. The crystal structure of the T4HNR complexed with the NADP(H) and pyroquilon (1JA9) revealed that fungicide pyroquilon binds with the crucial residues forming active site of the T4HNR protein and therefore, interrupt its functional mechanism. The fungicides that interact with the residues forming active sites or interact with the major residues that form the catalytic center of protein might have good results, for disrupting the functional aspect of proteins and therefore, would affect its possible biological roles. The metapocket server analyzed all the possible binding sites that might be occupied with the ligands, during the protein–fungicide interactions. The structural alignment unravelled the conserved T4HNR and replaced the key residues such as TYR^178^ with TYR^154^, LYS^182^ with LYS^158^, PRO^208^ with PRO^184^, THR^213^ with THR^186^, ASP^214^ with ASP^187^, MET^215^ with MET^188^, and TYR^223^ with TYR^196^ in the FOXG_04696. It was found that all the replaced residues in the FOXG_04696 were present in either major or another major (first two) cavities predicted by the metapocket server. In our previous studies, the structure of the functional domain of the proteins that belong to the *WRKY* gene superfamily members has been modeled for its qualitative and quantitative evaluation, to unravel the DNA–protein interaction studies, in a stimulus-specific manner in tomato (Aamir et al., 2017, 2018). In our results, the computational screening revealed the docking site and energy score values for all the 37 fungicides, to evaluate their efficacy against the FOL pathogen. The fungicide Famoxadone interacted with maximum energy (kcal/mol) with the key residues that constituted the prominent active site.

### Protein–Fungicide Docking and MD Simulations Analysis

The MD simulations of the protein–fungicide interaction reflected the time-dependent behavior of the biological complexes. The molecular docking and virtual screening revealed the two better fungicides including the Oxathiapiprolin and Famoxadone. The stability of the protein–fungicide docked complexes was measured at 50-ns MD simulations. It was found that both Oxathiapiprolin and Famoxadone disclosed the steadiness of the docked complexes, with an average potential energy of −113166.16 and −112628.96 kcal/mol, respectively. However, Famoxadone had comparatively better docking with XPG score of −3.30 kcal/mol (compared to Oxathiapiprolin; XPG score of −1.87 kcal/mol) along with lower values of RMSD and RMSF for protein–ligand contact, and better interaction within the particular specified docking site (within 4 Å binding site region of FOXG_04696). The lower XPG score −3.30 kcal/mol (more negative), and lesser RMSD and RMSF values for Famoxadone-FOXG_04696 (compared to Oxathiapiprolin_FOXG_04696) predicted the stability and reproducibility of the docking results, to find the crystallographic relevant and accurate binding pose.

We analyzed our docking results from two different molecular docking and virtual screening platforms including the Glide XP dock and an auto docked-based YASARA server. Finally, the complexes having good docking score, better *K*_d_ values, and accurate docking poses were further refined and rescored through the MD simulations, and MM/GBSA methods, to validate the top scored docking results. In each case, we found that the Famoxadone docked complex with FOXG_04696 had good docking score, with accurate docking pose, and was reliable and reproducible. The YASARA results evaluated the docking calculations based on YASARA score and dissociation constant (*K*_d_). Based on YASARA results, one could predict that fungicides having high YASARA scores and low *K*_d_ must bind with the receptor protein FOXG_04696 in a good docking pose. However, MD simulations analysis for these complexes (top docking score) were either failed to bind with the target receptor protein or were reported to be docked in an alternative conformation (other binding sites). Moreover, the fungicides having low dissociation constant (*K*_d_) values for the docked complexes such as Thiophanate methyl (*K*_d_ value 4.85), Trifloxystrobin (*K*_d_ value 4.86), Boscalid (*K*_d_ value 5.27), Pyraclostrobin (*K*_d_ value 6.69), and Isopyrazam (*K*_d_ value 6.84), despite of having good docking score and lesser *K*_d_ values, did not bind in a good docking pose in the MD simulations, or bound with sites in altered conformation (residues that were not involved in binding active sites, or other minor binding sites). By contrast, fungicides such as Carbendazim (*K*_d_ value 57.09), Cymoxanil (*K*_d_ value 22.45), Dithane (*K*_d_ value 976.17), Famoxadone (*K*_d_ value 2.43), Fluberidazole (*K*_d_ value 13.64), Metiram (Zineb) (*K*_d_ value 976.17), Pterostilbin (*K*_d_ value 24.47), Tebuconazole (*K*_d_ value 39.05), and Oxathiapiprolin (*K*_d_ value 1.86) bounded with some of the core residues that constituted, the major or minor binding sites of receptor protein in a good docking pose as revealed through YASARA. The YASARA score, *K*_d_ values, and MM/GBSA free energy binding values for Metiram (Zineb) and Dithane were found to be similar −55.63 (±0.38) as both share similar structure, and Dithane is the dimer unit of Metiram. The docking conformation of the Famoxadone and Oxathiapiprolin with the FOXG_04696 analyzed, and reported to be good from all the docking servers. Moreover, the MD simulations of Famoxadone and Oxathiapiprolin protein complexes showed better results with minimum interaction energies (Oxathiapiprolin followed by famoxadone), and also had lesser *K*_d_ (Oxathiapiprolin followed by Famoxadone). The MD simulations of Oxathiapiprolin complex had minimum interaction energy and *K*_d_ values, but the residues involved in interaction were non-significant, and were present beyond the binding sites (both major and minor) of target receptor protein. The residues that were found to be involved in Oxathiapiprolin binding during MD simulations were LEU^100^, VAL^103^, ILE^108^, LEU^112^, VAL^116^, TRP^146^, GLY^147^, VAL^148^, PRO^149^, ARG^150^, HIS^151^, ALA^152^, LEU^153^, SER^155^, ALA^156^, SER^157^, and ALA^160^. Moreover, Famoxadone had good binding affinity from all the platforms with having maximum residues from first binding site (major) including catalytic tetrad, of the FOXG_04696. The residues involved with the Famoxadone binding during MD simulations were GLY^13^, SER^15^, ARG^16^, GLY^17^, ILE^18^, GLY^19^, TYR^37^, VAL^38^, ASN^91^, SER^92^, GLY^93^, ILE^94^, GLU^95^, ILE^139^, SER^140^, SER^141^, ILE^142^, SER^143^, TYR^154^, LYS^158^, PRO^184^, LYS^185^, THR^186^, ASP^187^, MET^188^, TYR^189^, ALA^192^, ALA^193^, and TYR^196^ (exclusively forming major binding site of the receptor protein) (Supplementary Table S4).

It has been reported that multiple orientations (multiple different conformations adopted by ligands upon binding) could be involved in binding a ligand with proteins, and small conformational changes might have big effects on binding affinities (Mobley and Dill, 2009). Furthermore, these binding events are highly affected by multifarious factors, such as waters, ions, or cofactors, protonation state (changed protonation state on ligand binding), and/or conformational or solvation entropies that could have unexpected involvement and therefore, play unpredictable roles, in deforming the protein and ligands (Mobley and Dill, 2009). It has been demonstrated through several studies that the free energy calculations and MD simulations were done for refining and docking the docked complexes, starting from the docked poses, could be effective in increasing the accuracy of binding affinity predictions (Claussen et al., 2001; Andér et al., 2008). Numerous studies on molecular docking program have demonstrated that the computational screening for ranking the affinities of ligands binding to receptor proteins may results into a higher enrichment of active compounds than random screening (Stahl and Rarey, 2001; Wyss et al., 2003). However, they may suffer from sufficient false positive and false negatives, and are not sufficiently accurate to rank the compounds according to their binding affinities (Pearlman and Charifson, 2001).

In our results, we found the discrepancies in the ranking of ligand binding affinities from two different popular molecular docking programs (Glide XP and YASARA scores). In the YASARA binding energy function, the energy was calculated as the difference between the sum of potential and solvation energies of the separated compounds, and the sum of potential and solvation energies of the complex in the YAMBER3 force field. Thus, more positive YASARA score (difference) means higher affinity (Jakubík et al., 2013, 2015). In this context, Jakubík et al. (2015) analyzed the performance of four molecular modeling and docking programs (Autodock and Glide for docking; AutoDock binding energy function, Glide XP, Prime MMGB/SA, and YASARA binding function for pose scoring) in the pose evaluation of re-docked antagonists/inverse agonists to 11 original crystal structures of the aminergic G protein-coupled receptors (GPCRs), and found differences in the ranking of ligand binding affinities, from all the four molecular docking programs. In one study, Suenaga et al. (2012) reported that in the docking process, the top-scored docking pose does not always correspond to the optimal docking structure. Thus, the abilities to determine the optimal docking structure among multiple docking poses generated by the docking process, as well as to correctly rank the ligands according to their binding affinities, are important for successful computational screening. Furthermore, investigation of top posed protein–ligand interactions revealed substantial differences from actual crystallographic structures. In this way, the discrepancies observed in top docked poses and actual crystal structures, or bad ranking of top poses render all current docking and scoring schemes completely inefficient to rank-order drug leads for efficient drug optimization (Warren et al., 2006; Whalen et al., 2011; Suenaga et al., 2012; Ramírez and Caballero, 2016).

It has been reported that docking calculations performed through different servers and tools has several limitations such as a wrong binding site of target receptor protein, the choice of docking poses, high docking scores, but failed in MD simulations (Chen, 2015). Furthermore, sometimes MD simulations results revealed docking poses, that were actually unstable, but possess high docking score (Chen, 2015). In this regard, MD simulations could be deployed for calculating the conformational entropic changes upon receptor–ligand binding. This could be derived from time-dependent changes in atomic coordinates of the protein and ligand in both bound and unbound forms (Du et al., 2016). The stability and reliability of the docked complexes over the simulation time course provides a good indication for their reliability, accuracy, and stability as it was demonstrated that the unstable and incorrectly docked structures during MD simulations results into an unstable trajectories, that finally lead into disruption of the complex. By contrast, the realistic complexes provide stable behavior (Yunta, 2016). It has been shown that MD simulations are necessary for some systems to identify the correct binding conformations (Hou et al., 2011; Sakano et al., 2016). Therefore, MD can additionally be used to estimate the stability of a ligand–receptor complex proposed by molecular docking (Alonso et al., 2006). However, the more accurate prediction of binding affinity can be obtained through free energy calculations, dependent on thermodynamically important parameter that includes the interaction of protein and ligands in complexes, their interaction with water and other counter ions in unbounded formed, explicit inclusion of the solvent protein dynamics/flexibility (Du et al., 2016).

### Molecular Mechanics and Binding Energy Assessment

The binding energy calculations for molecular complexes could be calculated from MM/GBSA methods, which calculate binding free energies for molecules by combining molecular mechanics calculations and continuum (implicit) solvation models. With this view, the computational calculation for estimating the free binding energies are predicted from the difference between the free energy of each ligand bound to the protein and the free energies of the components of the complex, i.e., Δ*G*_binding_ = Δ*G*_complex_ - (Δ*G*_free_ _receptor_ + Δ*G*_free ligand_). The enthalpic contributions for docked complexes are assessed through molecular mechanics. MM/GBSA Δ*G*_bind_ negative value indicates stronger binding of the ligands with receptor protein. MM/GBSA (Δ*G*_bind_) can be expected to agree reasonably well with ranking based on experimental binding affinity. The results obtained for binding energies calculations of the protein–ligand interactions through MM/GBSA calculation were reported to be highly reproducible and stable (Genheden and Ryde, 2015), and independent of solvation of the receptor protein, selection of alternative conformation in the starting crystal structure, uncertainty in protonation and conformation of various groups (if employed with care) (Genheden and Ryde, 2015). The calculations set up by different groups and procedures are likely to give similar results, in spite of the many more or less arbitrary choices made during the setup (Genheden and Ryde, 2015). Furthermore, MM/GBSA provides more rigorous solutions for better prediction of reliable and accurate binding positions, and to estimate the free energies of the bound molecular complexes (Zhang et al., 2017). This could be attributed due to the fact that MM/GBSA based scoring is physics-based term, which contains explicit terms for hydrophobic, *V*_DW_, or solvation components. By contrast, other docking and scoring based programs calculate an empirical scoring function likewise machine based learning procedure, and with having no relevance with other physical parameters. The binding energy was calculated as the difference between the MM/GBSA energy of the complex and the sum of MM/GBSA energies of the unliganded receptor and the free ligand. It has been found that the top docking ranked poses are the lowest ranked poses using MM/GBSA rescoring, that indicates the rescoring of few top poses, if binding could not be determined through docking programs or binding is nonspecific. In many studies it has been well demonstrated that MM/GBSA approach is most accurate and reliable for ranking (“scoring”) the efficacy/affinities of a ligand binding to the receptor proteins in the protein–ligand docked complexes (Singh and Warshel, 2010; Sun et al., 2014; Wright et al., 2014; Genheden and Ryde, 2015; Maffucci et al., 2018). MD simulations analysis therefore, could be employed for accurate ranking of ligands following the post docking program in terms of their binding affinities (Okimoto et al., 2009; Chen, 2015). Recently, MM/GBSA based on short MD simulations has been employed for prediction of the accurate poses among the generated docking poses (Terayama et al., 2018).

Overall, one important conclusion from our study revealed that, docking studies must be harmonized with MD simulations, as MD simulations provide core information to complement the docking prediction, and unravelled the docking poses that were actually unstable. The MD simulations equilibrate the system to achieve a stable conformation. If the initial structure was energetically unstable, the system appropriately changes the conformation in subsequent MD simulations (Sakano et al., 2016). Moreover, MD simulations consider the natural motion of protein whereas docking usually utilizes a single structure obtained by experiment. The binding energy predictions were highly correlated with a correlation coefficient *r*^2^ = 0.335 for the selected protein–fungicide docked complexes, and reported Famoxadone and Oxathiapiprolin having better binding efficiency with FOXG_04696 than other fungicides.

The selected fungicide (Famoxadone) was further evaluated for *in vitro* inhibitory test against the FOL pathogen. It was found that the selected fungicide was good enough as the mycelial growth was found to be inhibited at every increased concentration at an increased time interval. The *in silco* toxicity assessment tools further predicted the toxicity assessment of the fungicide and was found to be acceptable for environmental disposition, and could be used safely against the FOL pathogen for controlling the vascular wilt disease.

It is well known that fungal SDRs are large family enzymes and play a crucial role in various metabolic processes, their functional characterization in the FOL pathogen, is an interesting approach. The predictive function of the desired protein could be useful in understanding the virulence mechanism and resistance of the FOL pathogen to target fungicides. Moreover, this protein could be better deployed in structure-based drug design and catalysis. The functional relevance of the FOXG_04696 (T4HNR like) is not quite understood. In this context, we could predict that the hypothetical protein FOXG_04696 might have possible functional role in secondary metabolic process (3-oxoacyl-[acyl-carrier protein] reductase), versicolorin reductase (melanin pigment biosynthesis), or play crucial role in the FOL virulence (alcohol dehydrogenase) (based on the results of significant hits of Blast-p annotation). The possible functional relevance of the *in silco* predicted protein could be deduced and determined experimentally using mutant analysis and genetic complementation studies. The data from our study will drive future experimentation for determining the predictive function of this protein in the FOL pathogen.

## Conclusion

The present research work provides an insight into the structural, functional, and dynamical aspects of fungal SDR (T4HNR like) in the FOL pathogen. The computational modeling of protein 3D structures, with high accuracy and functional characterization, revealed the core information regarding the homology and conservation of SDRs among the closely related fungal taxonomic groups. The fungal SDRs play a crucial role in various metabolic processes including biosynthesis of melanin and other pigments, mycotoxin biosynthesis, secondary metabolism, fungal defense response, and fungal pathogenicity; these enzymes could be deployed as novel targets, for the discovery of novel agrochemicals against the phytopathogenic fungi. We reported the interaction of Famoxadone with FOXG_04696 (T4HNR like) with best protein ligand contacts through the core residues from major binding site of receptor protein. The protein–ligand interaction also targeted the functional residues that constituted the (active sites) and in a good docking pose with least binding energy. Interestingly, the X-ray diffracted crystal structures or NMR-derived solution structures, of protein–ligand complexes, could be used for interaction studies with unknown hypothetical proteins. Moreover, the inhibitors discovered through hierarchical *in silico* screening approach (pharmacophore modeling and molecular docking) could be employed for comparative binding studies of an experimentally derived molecular complex, with unknown hypothetical protein and novel ligands. The experimental data available for protein–ligand interaction at good resolutions could help in analyzing the other relevant proteins and complexes for the better modulation of their functional activity in a more efficacious and reliable manner. The computational screening for getting a novel inhibitor (fungicide) followed by *in vitro* assessment, could be useful to develop commercial formulations either alone or in combination with other better fungicides, or used with other integrated approaches, for the better management of the Fusarium wilt disease.

## Author Contributions

MA and VS conceived the idea and planned the experiments. MA performed all the experiments, did the computational analysis of results, and finally prepared and wrote the manuscript. VS assisted in the computational analysis of results. MM performed the *in vitro* experimental work, and also helped in computational analysis of the results. MD assisted MM in *in vitro* experimental work, and also helped MA in the computational analysis of the results. SKK, SPK, and AU analyzed the MD simulations analysis of the protein–fungicide interactions. MA, SKK, and MD prepared the final version of manuscript. SS, RU, and AU assisted in manuscript writing, data validation, and supervised the work throughout the study. All authors revised and approved it for publication.

## Conflict of Interest Statement

The authors declare that the research was conducted in the absence of any commercial or financial relationships that could be construed as a potential conflict of interest.

## Footnotes

1 http://blast.ncbi.nlm.nih.gov/Blast.cgi

2 http://www.megasoftware.net/

3 http://circos.ca/

4 http://prosite.expasy.org/scanprosite/

5 https://www.ebi.ac.uk/interpro/interproscan.html

6 http://www.softberry.com/

7 http://www.cathdb.info/

8 http://www.cathdb.info/search/by_funfhmmer

9 http://revigo.irb.hr/

10 http://cello.life.nctu.edu.tw/cello2go/

11 https://www.ncbi.nlm.nih.gov/Structure/cdd/wrpsb.cgi

12 https://projects.biotec.tu-dresden.de/metapocket/

13 http://string-db.org/

14 http://www.rcsb.org/pdb/

15 http://www.ebi.ac.uk/pdbsum/

16 http://mordred.bioc.cam.ac.uk/~rapper/rampage.php

17 https://prosa.services.came.sbg.ac.at/prosa.php

18 http://services.mbi.ucla.edu/ERRAT/

19 http://www.scfbio-iitd.res.in/software/proteomics/protsav.jsp

20 http://vadar.wishartlab.com/

21 http://eds.bmc.uu.se/eds/australis.php

22 http://www.caspur.it/PMDB

23 http://fafdrugs3.mti.univ-paris-diderot.fr/

24 http://lmmd.ecust.edu.cn/admetsar1/predict/

25 http://www.scfbio-iitd.res.in/software/drugdesign/lipinski.jsp

## Abbreviations

DHN: 1,8-dihydroxynaphthalene
DOPE: discrete optimized protein energy
GLIDE: grid-based ligand docking with energetics
MM/GBSA: molecular mechanics generalized Born surface area
PMDB: protein modeling database
ProSA: protein structural analysis
ProTSAV: protein structure analysis and validation
RAMPAGE: Ramachandran plot analysis
RMSD: root mean square deviation
RMSF: root mean square fluctuation
SDR: short-chain dehydrogenase/reductases
T3HNR: 1,3,8-trihydroxynaphthalene reductase
T4HNR: 1,3,6,8-tetrahydroxynaphthalene reductase
UniProtKB: Universal Protein Resource Knowledgebase
VADAR: volume area dihedral angle reporter
OPLS: (optimized potentials for liquid simulations).
