# Supplementary material for: In silico Prediction, Characterization, Molecular Docking, and Dynamic Studies on Fungal SDRs as Novel Targets for Searching Potential Fungicides Against Fusarium Wilt in Tomato
Source: Front Pharmacol. 2018 Oct 22;9:1038. doi: 10.3389/fphar.2018.01038 (PMC6204350; doi:10.3389/fphar.2018.01038)
Supplement: Supplementary file 1 [file Table_1.DOCX]

**All Supplementary Information with Figure Legends and Tables**

**
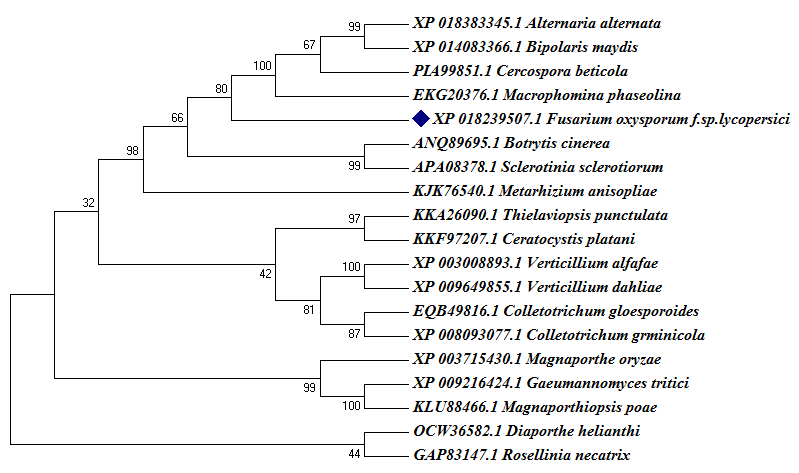
**

**Figure S1** Maximum parsimonious based phylogenetic tree showing the evolutionary conservation and functional diversification of fungal SDRs across the related taxonomic group. The trre was generated at 1000 bootstrap replications


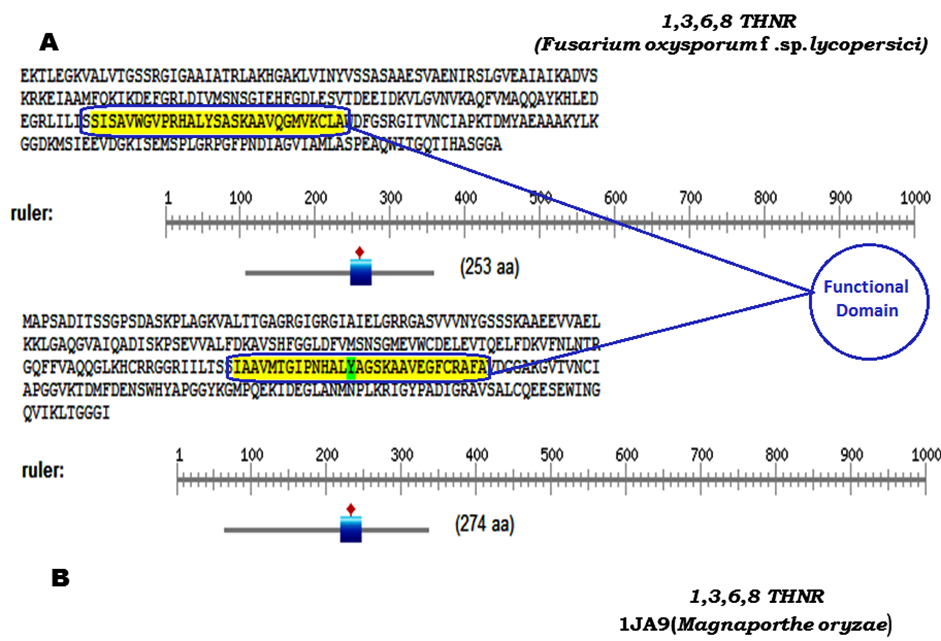


**Figure S2** PROSITE Scan Results showing the presence of conserved Domain in both 1JA9 and FOX_04696 . Comparative assessment of protein functional domain in FOXG_04696 and template model Protein (1JA9) as revealed by ExPASy-PROSITE tool. The yellow highlighted region predicts the functional domain of FOL-T4HNR and belongs to Short chain dehydrogenase reductase family (SDR)

**
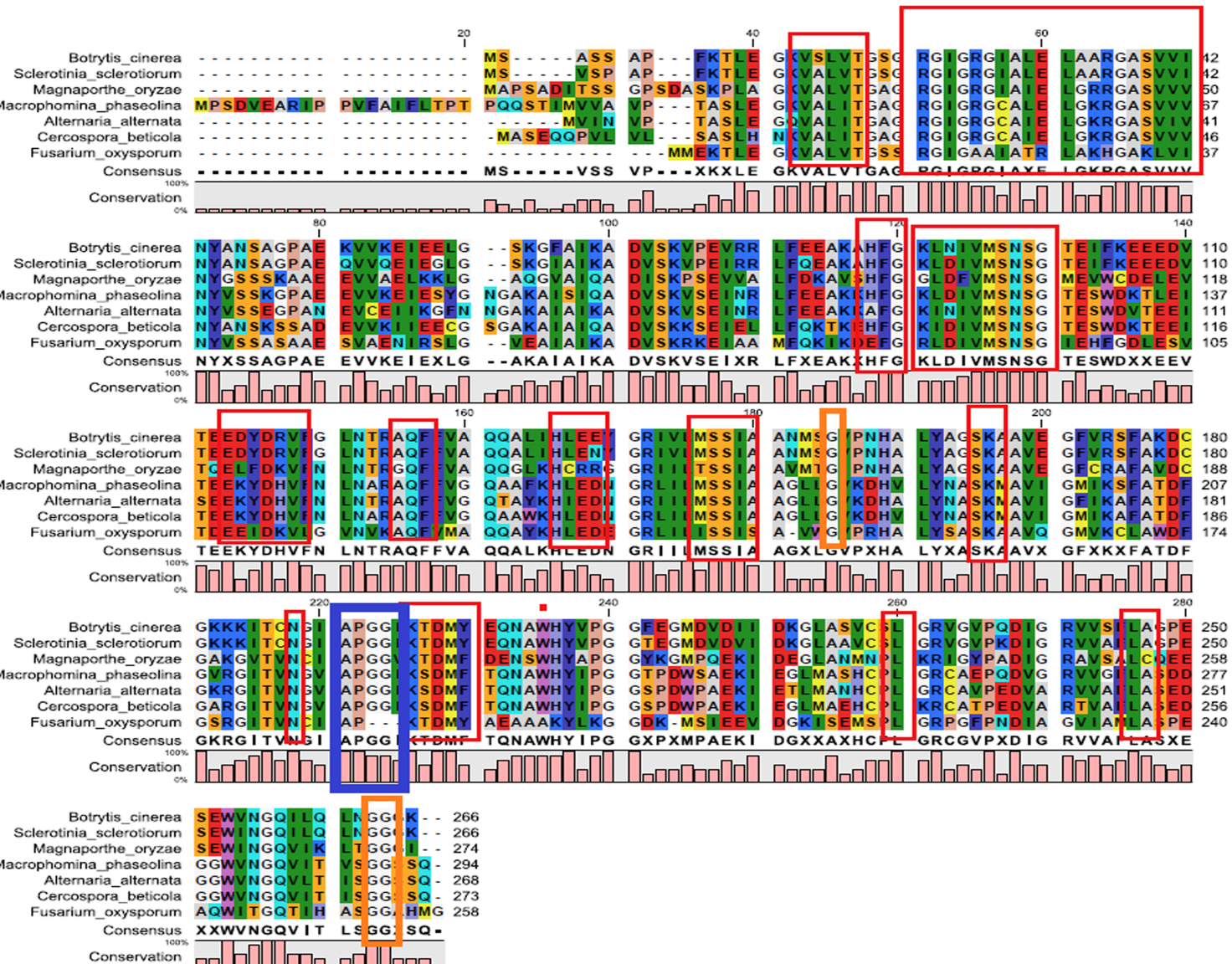
**

**Figure S3** Multiple sequence alignment results as obtained through CLC BIO work Bench tool. The figure represents the presence of conserved and consensus sequences for T4HNR protein in major phytopathogenic fungal taxa. The red square region denotes the position of conserved residues. The blue (bold) coloured square represent the absence of Glycine residues in FOX_04696 (present in all other members). The yellow-orange square represents the exclusively conserved glycine rich motif in all fungal taxonomic groups.

**
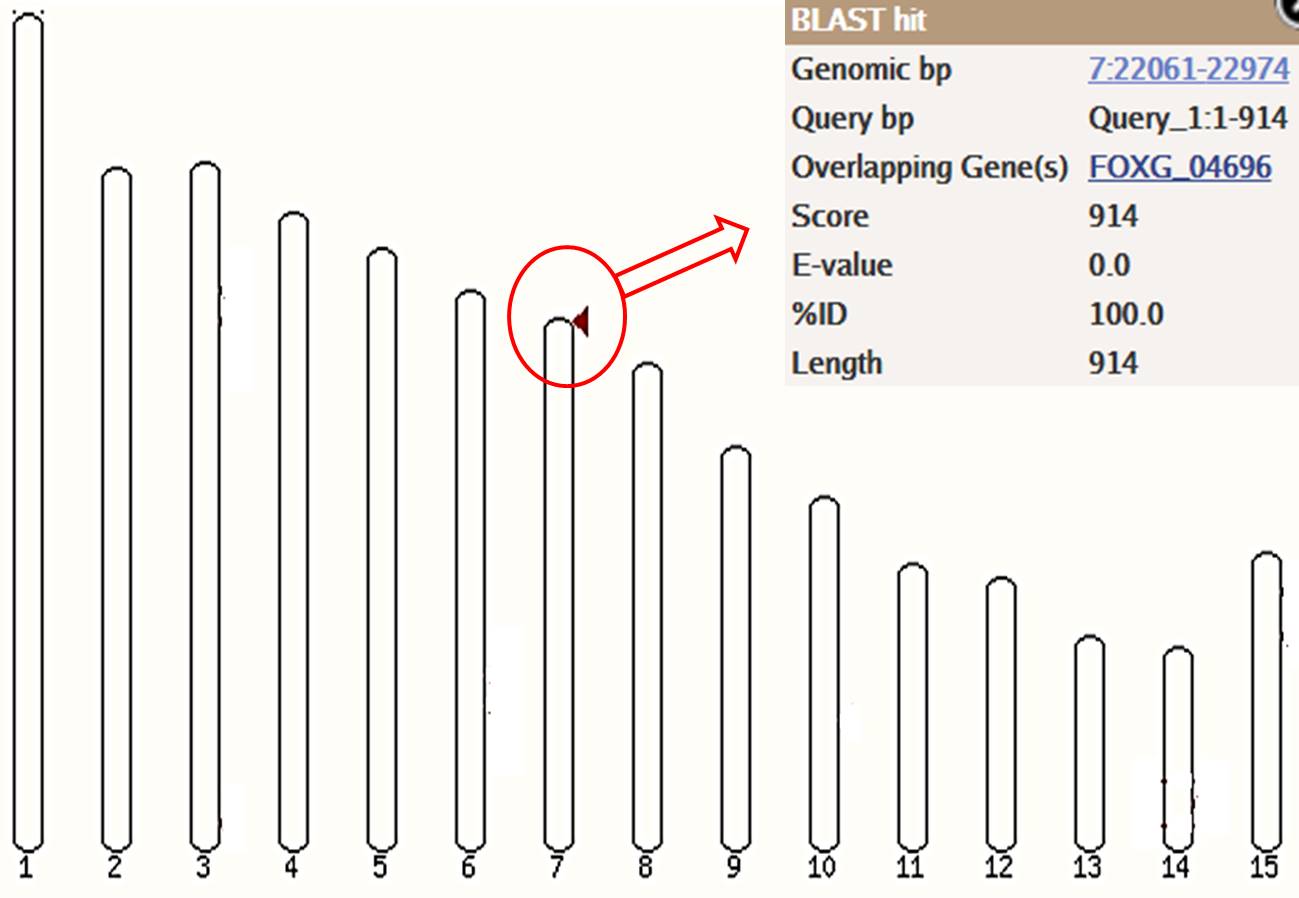
**

**Figure S4** Chromosome map for locating the position of FOXG_04696 protein. The gene encoding for FOXG_04696 has been found to be located on chromosome 7 at position in between the 22061-22974(with percent identity 100%; E-value 0.0) and revealed through Ensemble-Blast tool**.**

**
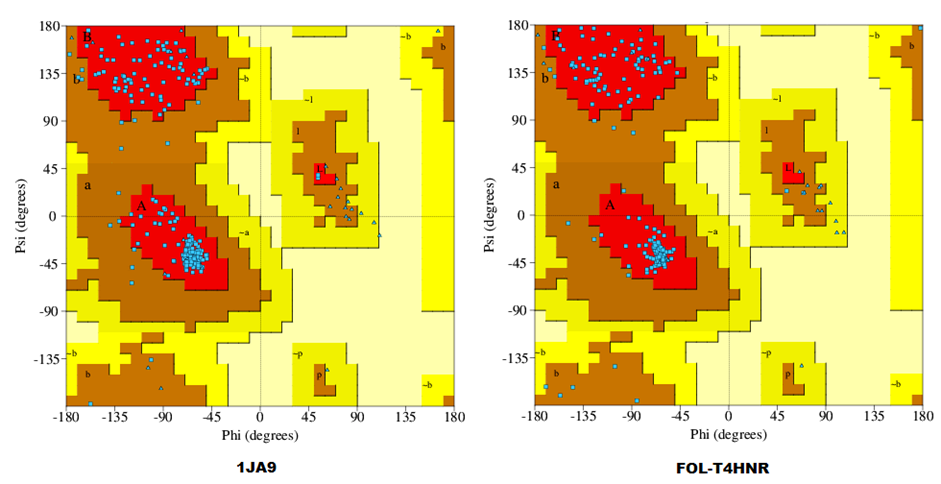
**

**Figure S5** The comparative qualitative assessment of the predicted (FOL-T4HNR) and X-Ray solved model (template) for the stereo chemical spatial arrangement of amino acid residues and were computed with the PROCHECK server. Most favored regions are colored red, additional allowed, generously allowed, and disallowed regions are indicated as yellow, light yellow and white fields, respectively.

**
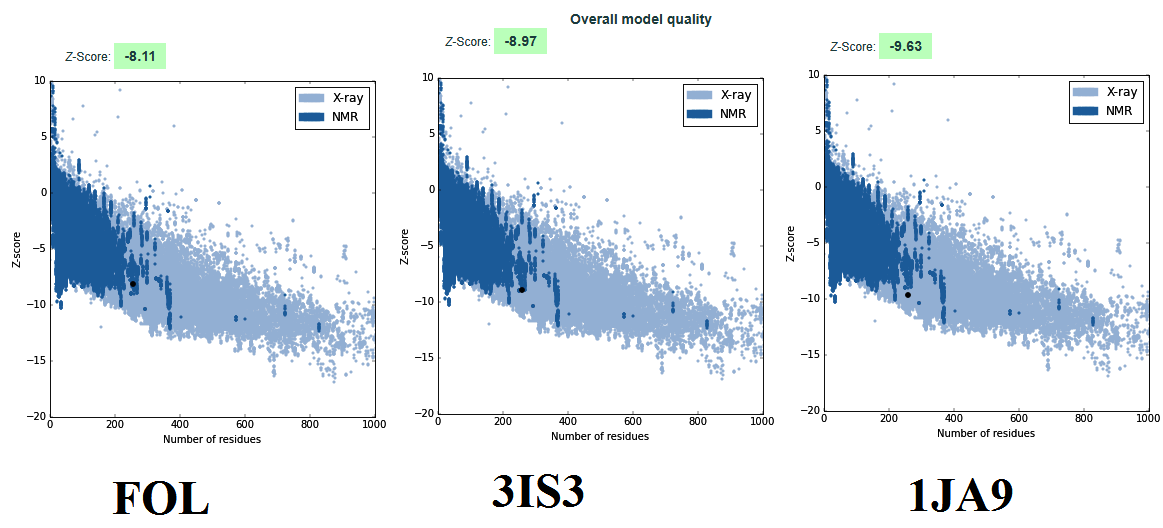
**

**FigureS6** Qualitative evaluation using ProSA webserver, which generates a plot measuring the structural error at each residue in the protein and calculate the overall score for quality measurement. The ProSA score for FOLT4HNR and 1JA9 were found closer to the native structures.

**
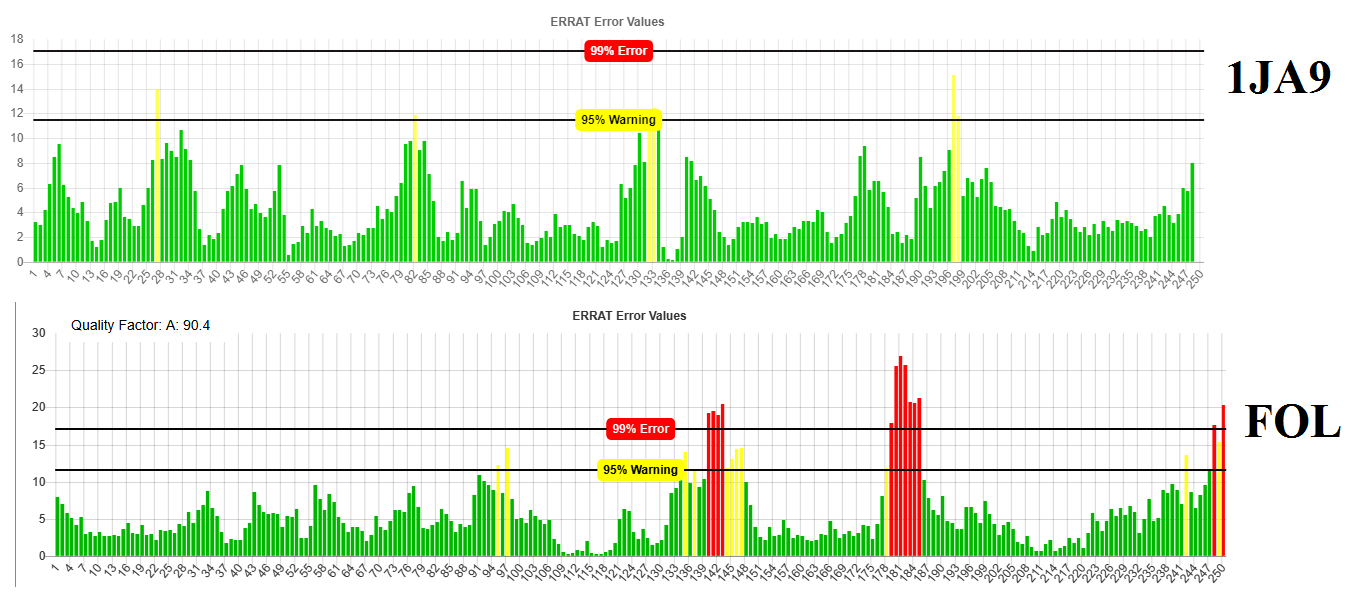
**

**ERRAT Scores**

**Figure S7** Qualitative assessment of the predicted model FOXG_04696 and template (1JA9) based on ERRAT score values

**
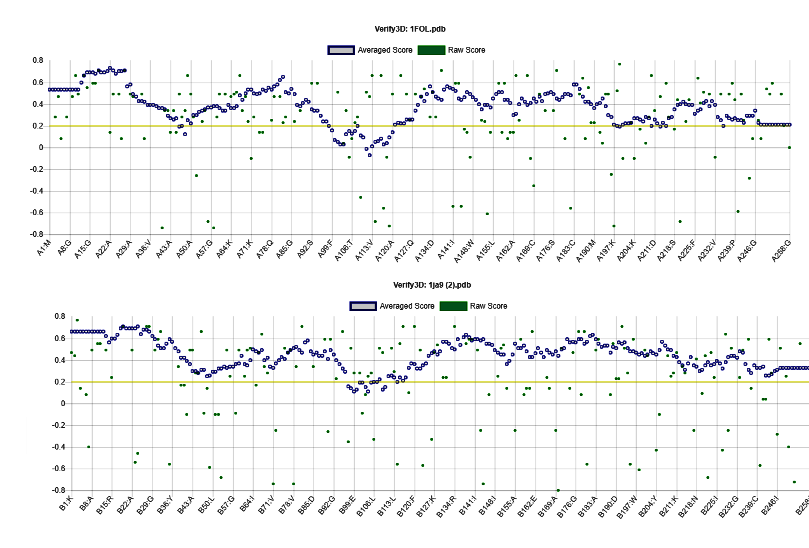
**

**VERIFY 3D Results**

**Figure S8** Qualitative assessment of the predicted model FOXG_04696 and template (1JA9) based on Verify-3D score values

**FOXG_04696**

**
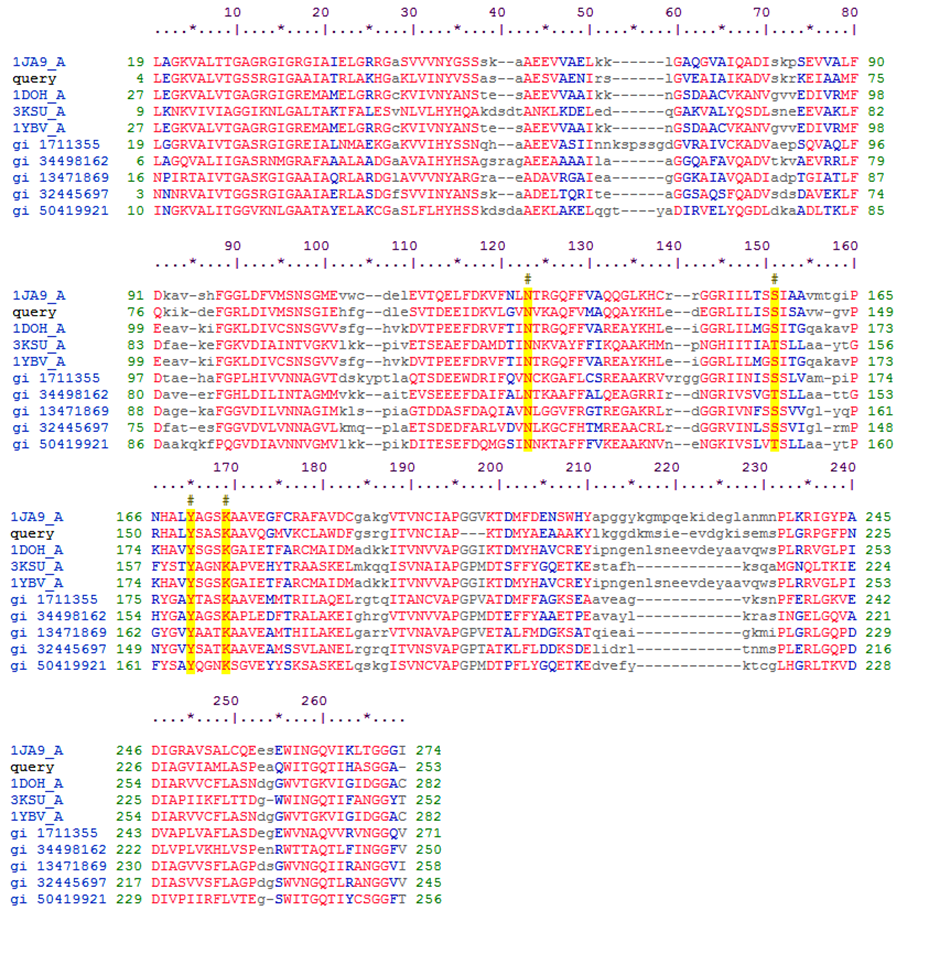
**

**Figure S9** The active site prediction for short chain dehydrogenase reductases (FOL-T4HNR) as revealed through Conserved domain database (CDD) server. The yellow coloured residues represent the residues forming active sites and play crucial function in enzymatic catalysis of napthol reduction reactions.

**
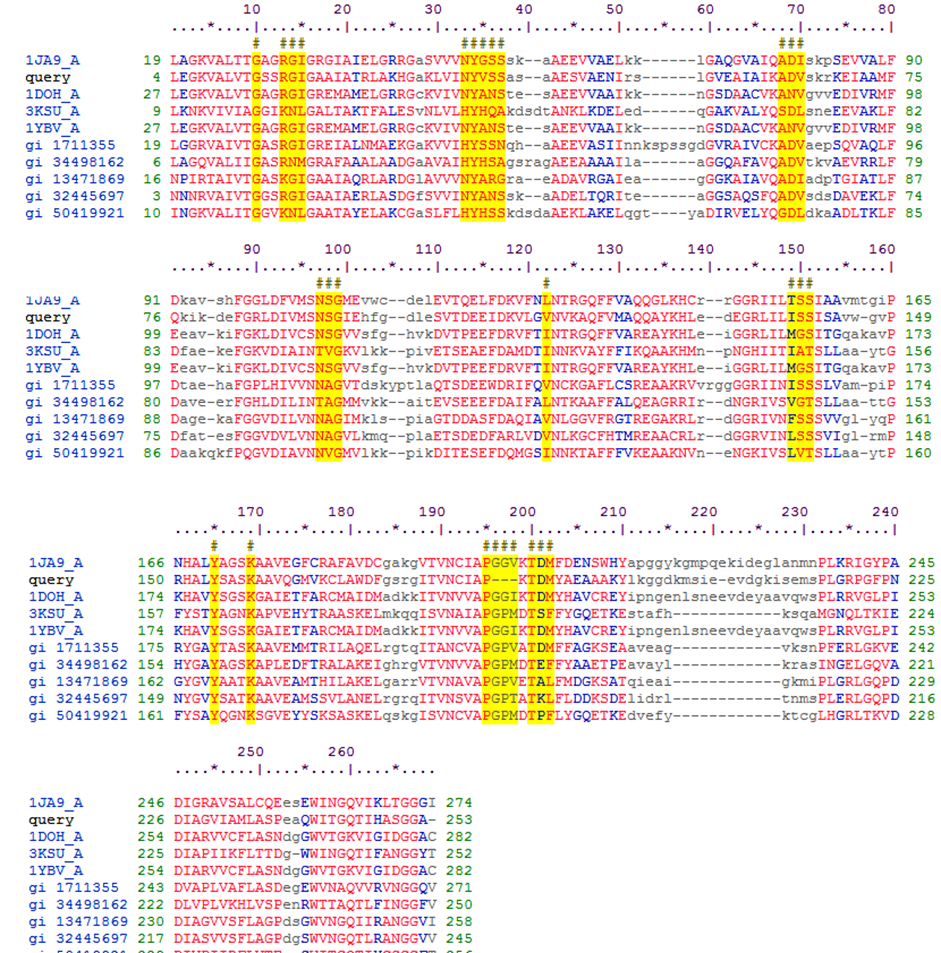
**

**Figure S10.** The prediction of chemical (NADP) binding sites in FOL-T4HNR as revealed through CDD blast server. The yellow region represents the conserved motifs having crucial role in binding with NADP. The highlighted residues were found to be conserved in all the SDRs


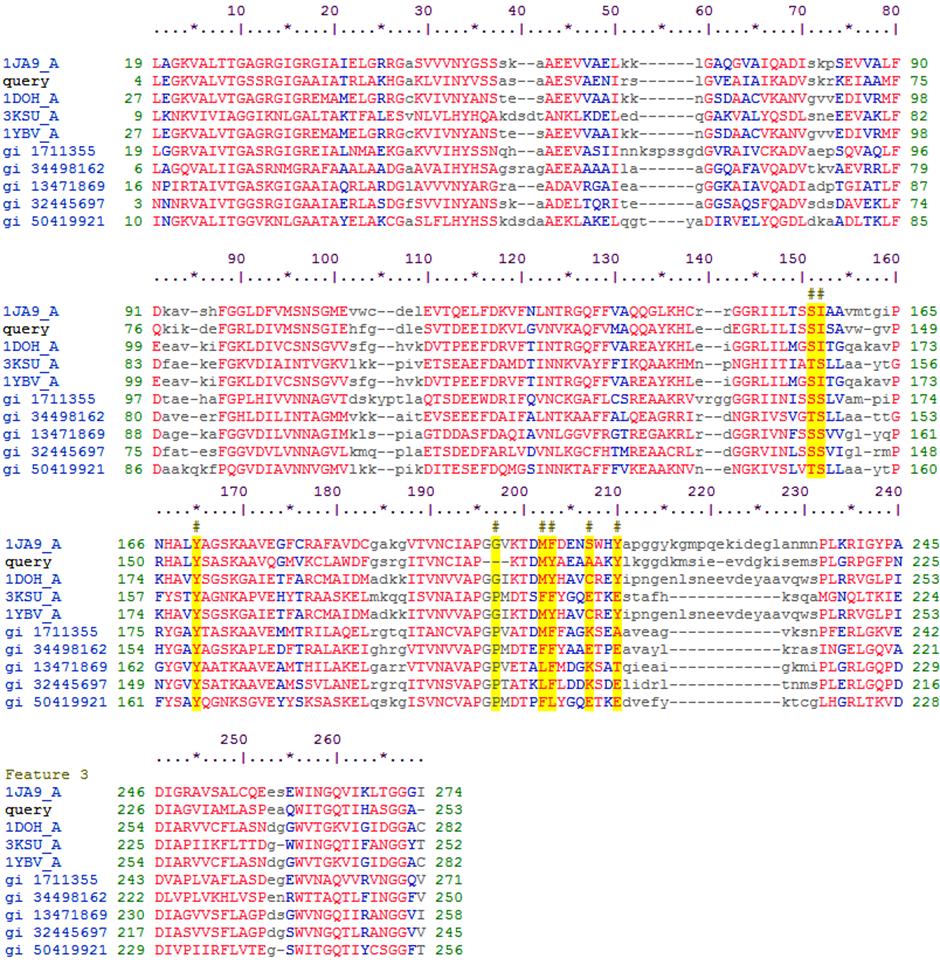


**Figure S11** The prediction of chemical (substrate) binding sites in FOL-T4HNR as revealed through CDD blast server. The yellow region represents the conserved residues having crucial role in binding with ligands. The highlighted residues were found to be conserved in all the SDRs

**BIOLOGICAL PROCESSES**

**
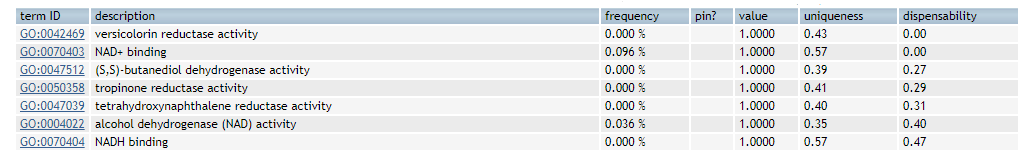

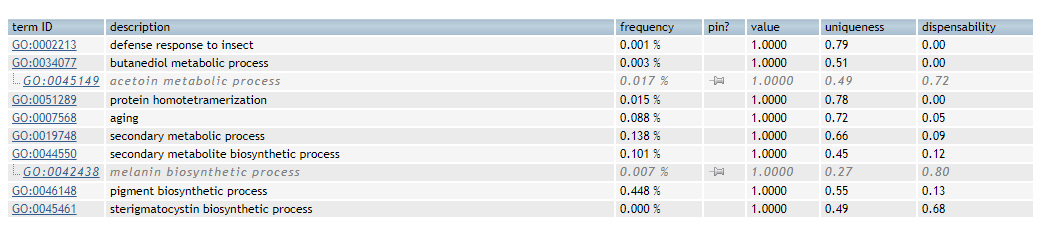
**

**MOLECULAR FUNCTION**


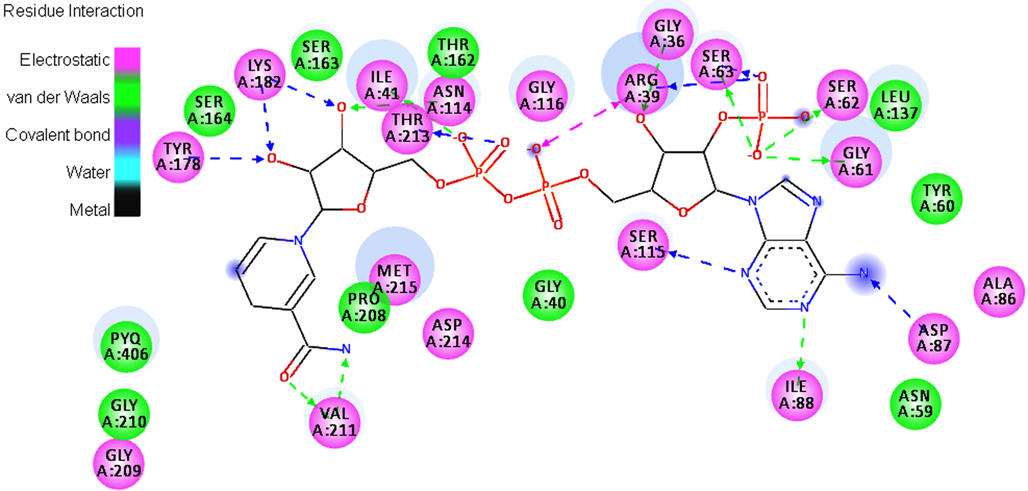


**Figure S13** The active sites residues showing interaction of fungicide pyroquilon with the template protein (T4HNR of *Magnaporthe grisea*). The figures showed all the possible type of interactions (electrostatic, Vander wall and covalent) with the template protein involving the groups from H bond donor or acceptor.

**
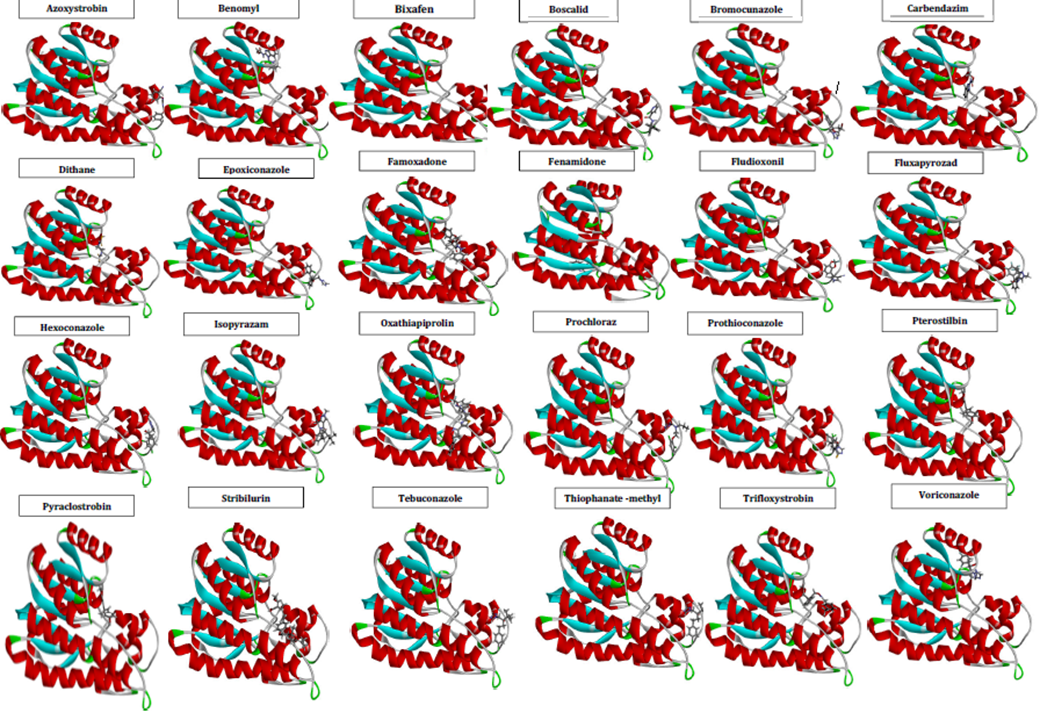
**

**Figure S14** Protein Docking interaction results as obtained through YASARA and were visualized in DS Visualization module. It is noteworthy that the entire different fungicides bind at separate binding site or cavities formed inside the protein. The fungicide (famoxadone) binds with the residues forming major binding site.


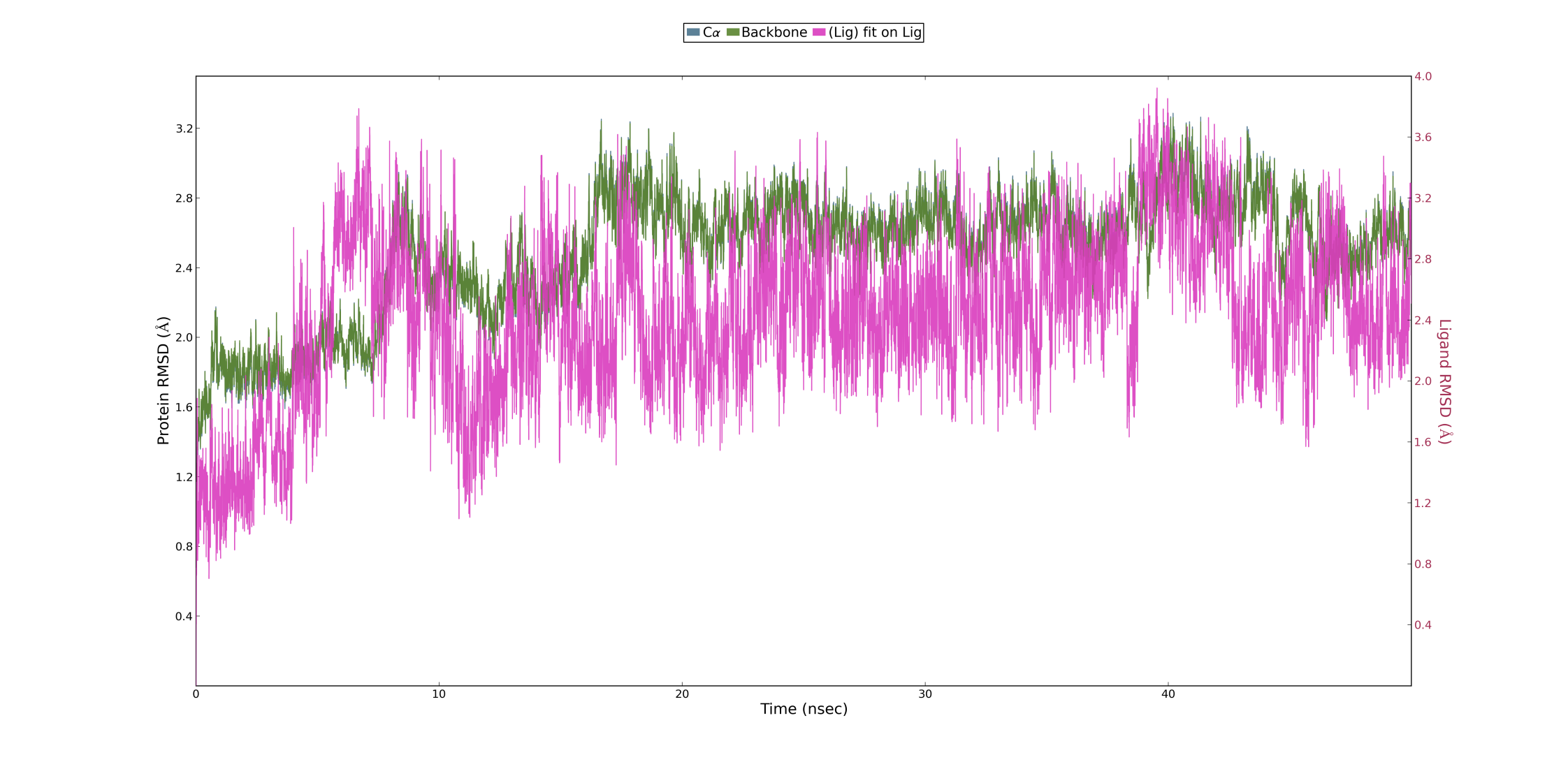


A


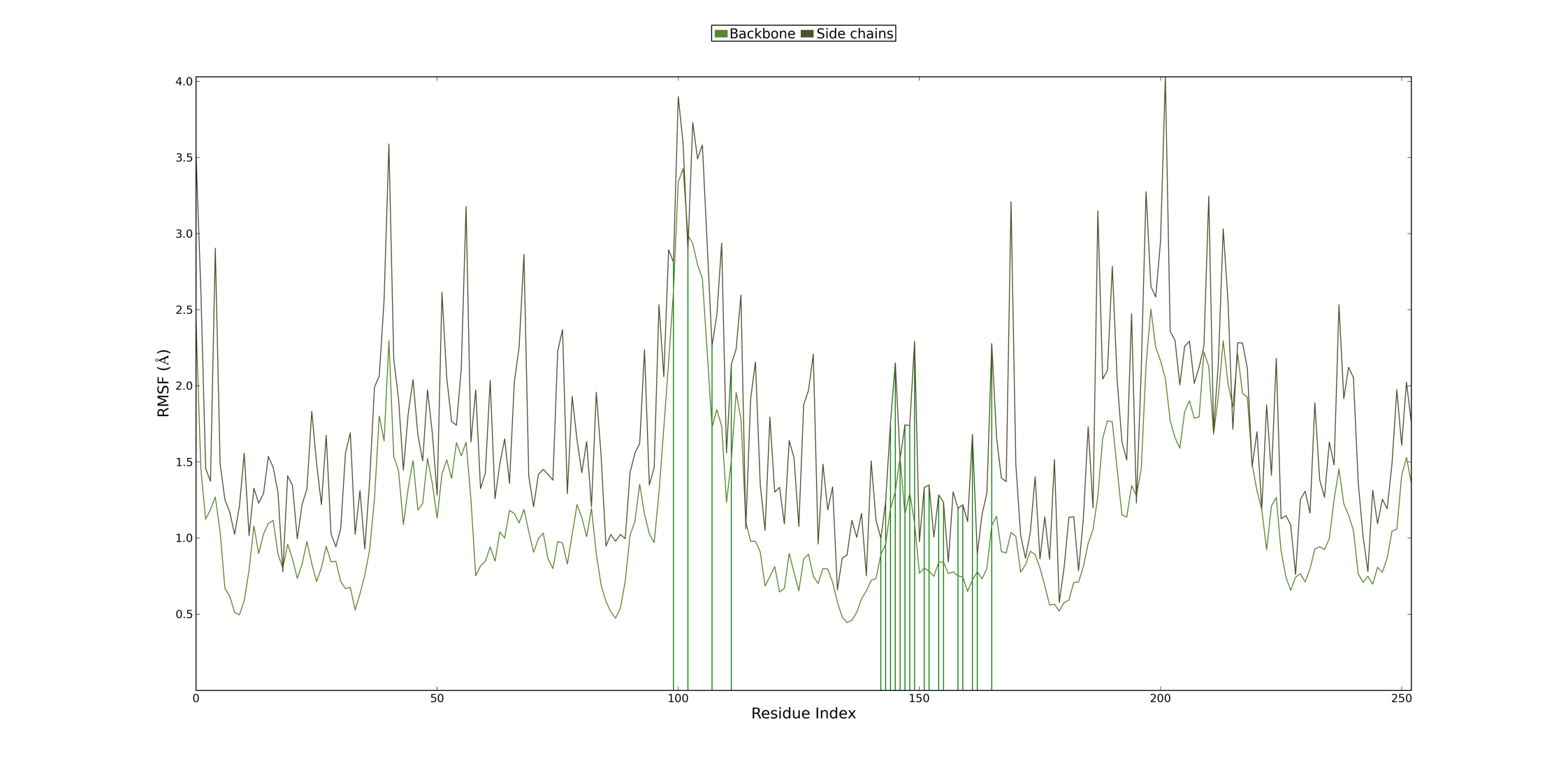


B

C


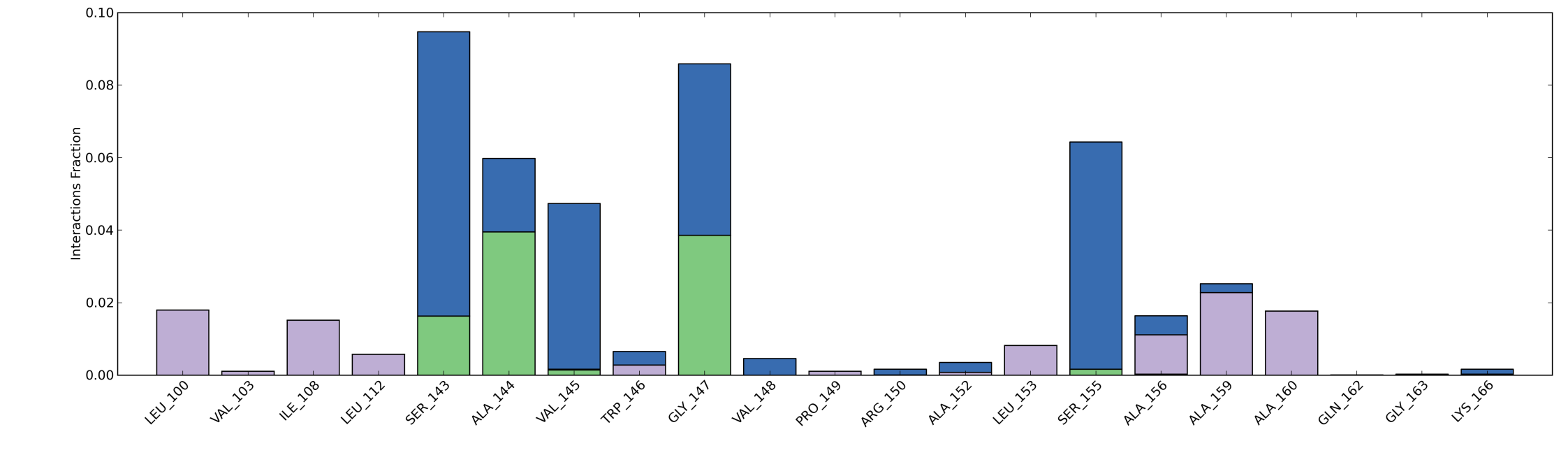


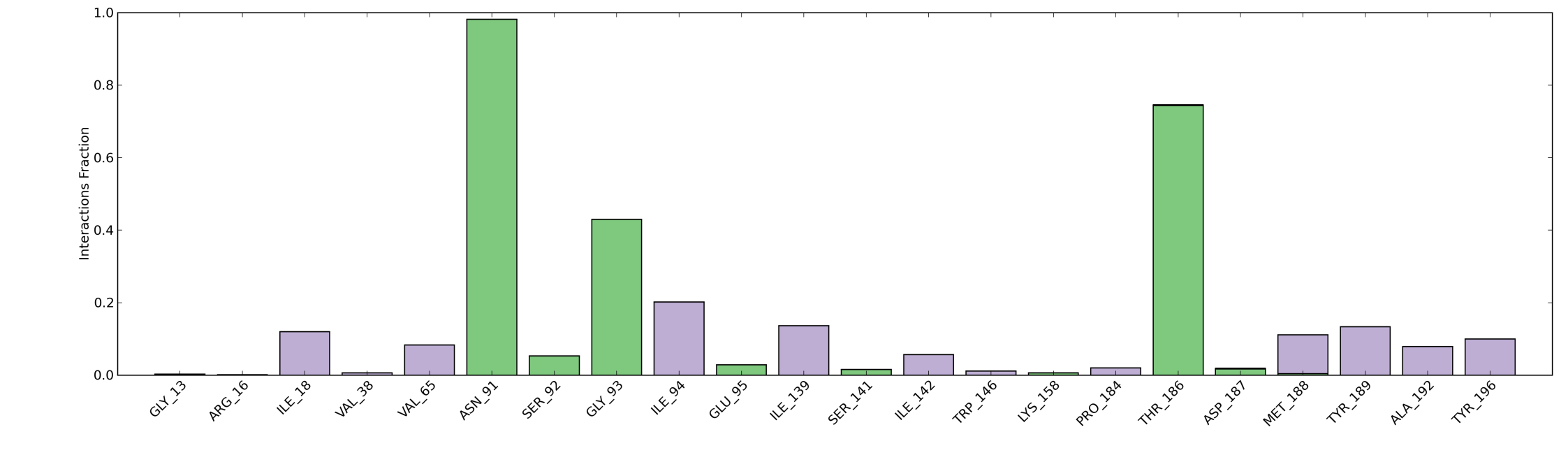


D


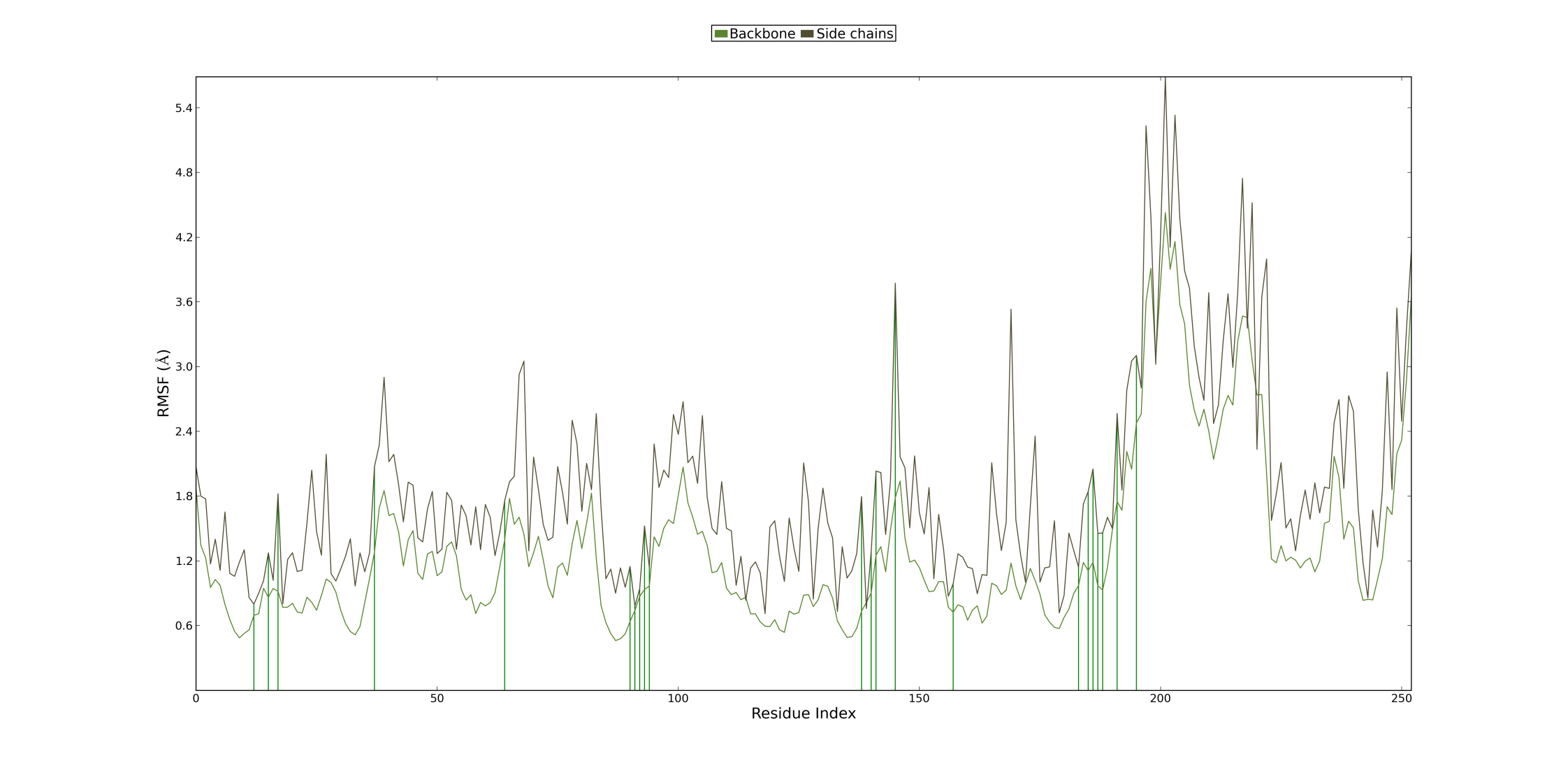


E

**Figure S15.** A.Trajectories representing energies are potential energy of the oxathiapiprolin-T4HNR complex during 50000ps or 50ns MD simulations analysis. (B)Protein ligand contact Map for Oxathiapiprolin and FOXF_04696. (C) RMSF plot values for protein ligand interaction (D). Protein ligand contact Map for famoxadone and FOXF_04696 showing the active site residues interaction fraction with famoxadone in 50 ns molecular dynamics simulationss. (E). RMSF plot values for famoxadone-FOXG_04696 complex.


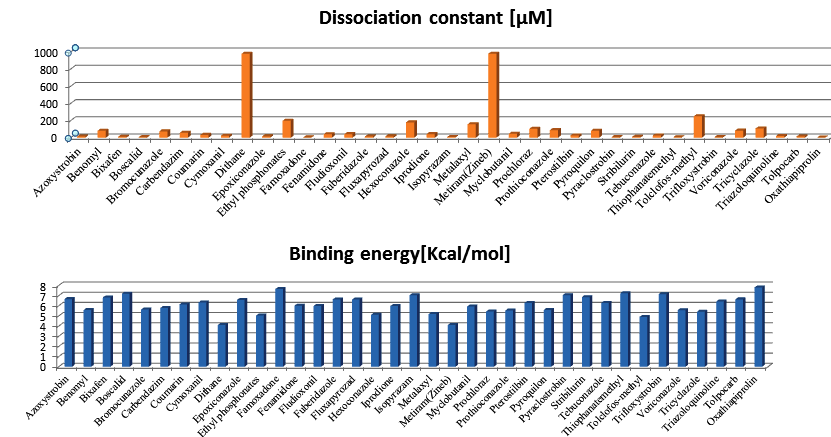


**Figure S16.** Evaluation of the protein ligand interaction through the YASARA. The YASARA calculated the binding energy and dissociation constant (K_d_) for the protein-ligand docked molecular complexes. In our result, the Oxathiapiprolin was found to have the maximum binding energy in positive values (where more positive energies indicate stronger binding, and negative energies mean no binding) with the least K_d_ followed by the Famoxadone.

**Table S1** **(Data given in separate MS-world file**)

Qualitative and Quantitative assessment of the lab derived structures and X-ray diffracted protein structure and computationally predicted models for receptor protein FOXG_04696 in terms of their qualitative assesment score covered significant residues

**Table S2. (Data given in separate MS-world file)**

Prediction of putative active sites that could be associated with template protein and our predicted protein. The first three binding sites have been shown and of which the first binding site represent the major binding site (active site)

**Table S3** **(Data given in separate MS-excel file)**

Protein- ligand docking using YASARA tool. The binding energies, dissociation constant and the contact receptor residues involved have been shown for each fungicide. The Oxathiapiprolin – FOXG_04696 complex was found to have maximum binding energy with least Kd values. The famoxadone_FOXG_04696 complex had second higher binding energy after Oxathiapiprolin –protein complex. The results obtained through YASARA proof the potential efficacy of Oxathiapiprolin and famoxadone over other fungicides (Note* YASARA calculate the binding energy in positive score where positive energy means stronger binding and negative energy means no binding)

**Table S4 (Data given in separate MS-word file)**

The GLIDE docking results showing the residues involved in protein –fungicide docked complexes. Oxathiapiprolin although had higher YASARA score with least K_d_, gave similar results with MD simulations but the residues involved were different as those observed from YASARA, and beyond the docking pose of receptor protein. Famoxadone interacted with the highest number of residues involved during interaction were from major binding site including the catalytic tetrad of FOXG_04696. The residues highlighted with yellow colour were reported from first (major) binding site (metapocket results). The residues shown in red colour were from second (minor binding site). The residues marked with green color were common in both major and minor binding site. The purple pink colour showed those residues that were beyond the major and minor binding site cavities(not present in any binding site).

**Table S5. (Data given in separate MS-excel file)**

The protein-protein interactive association network as revealed through the STRING server. The functional annotation, accession ID and interacting score values for both first and second shell of interactors that form mutual interactive associative network along with FOXG_04696. The higher value of score indicate the more frequent interaction exist between two associated proteins. The functional annotation has taken only for partners that interact with highest confidence.
