# Supplementary material for: In silico Prediction, Characterization, Molecular Docking, and Dynamic Studies on Fungal SDRs as Novel Targets for Searching Potential Fungicides Against Fusarium Wilt in Tomato
Source: Front Pharmacol. 2018 Oct 22;9:1038. doi: 10.3389/fphar.2018.01038 (PMC6204350; doi:10.3389/fphar.2018.01038)
Supplement: Supplementary file 2 [file Table_2.DOCX]

| S.No | **Protein Name** | **ERRAT**  **score** | **ProSA**  **Z score** | **Most Favored (%)** | **Additionally allowed**  **(%)** | **Outlier residues (%)** |
| --- | --- | --- | --- | --- | --- | --- |
| 1 | Crystal structure of 1,3,6,8 tetra hydroxynaphthalene reductase(T4HNR)  (*Magnaporthe grisea*)  (PDB ID : 1JA9)  (template1) | 97.5 | -9.63 | 98.4 | 1.6 | 0.0 |
| 2. | Crystal structure of 17beta-hydroxysteroid dehydrogenase (Apo form) *Cochliobolus lunatus*  (PDB ID : 1JA9)  (template2) | 99.2 | -8.97 | 98.0 | 1.6 | 0.4 |
| 3. | Short chain dehydrogenases/reductases (*Fusarium oxysporum* f.sp.*lycopersici*)  ( predicted model) | 90.4 | -8.11 | 97.7 | 1.6 | 0.7 |

**Table 1.** Comparative assessment of the experimentally solve protein structure (X-Ray diffraction) with the predicted model protein (FOXG_04696) based on their qualitative values.
