# Supplementary material for: In silico Prediction, Characterization, Molecular Docking, and Dynamic Studies on Fungal SDRs as Novel Targets for Searching Potential Fungicides Against Fusarium Wilt in Tomato
Source: Front Pharmacol. 2018 Oct 22;9:1038. doi: 10.3389/fphar.2018.01038 (PMC6204350; doi:10.3389/fphar.2018.01038)
Supplement: Supplementary file 3 [file Table_3.DOCX]

| **S.No** | **1,3,6,8 Tetra hydroxynapthalene reductase(T4HNR)**  ***Magnaporthe* *oryzae*(1JA9)** | **FOXG_04696**  **(*Fusarium oxysporum* f.sp.*lycopersici*** |
| --- | --- | --- |
| Binding Site 1 | ASN59 GLY61 ALA86 ASP87 ILE88SER115 GLY36 TYR60 GLY38 ARG39 SER63 SER62 LEU137 GLN85 THR35 ASN114 SER64 ALA66 SER89 GLY116 GLY42 ILE41 GLY40 THR162 LYS182 THR213 SER163 PRO208 TYR178 ILE206 MET215 ASP214 LYS133 ASN136 SER164 MET117 GLY209 VAL211 ILE255 GLY210 GLU118 PHE216 ILE165 LYS212 ALA166 ILE282 TYR223 ASN219 MET169 GLU218 SER220 VAL119 LEU240 TRP221 ILE45 MET112 ILE172 HIS222 ALA224 GLY281 | ILE^94^ GLU^95^  TYR^154^ THR^186^ MET^188^ TYR^189^ ILE^18^  ASN^91^ ILE^139^ SER^140^ SER^141^ LYS^158^ PRO^184^ GLY^93^ LYS^185^ SER^92^ ASP^187^  ARG^16^ GLY^17^ GLY^19^  TYR^196^  GLY^13^  SER^143^ ILE^142^ ALA^192^ SER^15^ VAL^38^ SER^40^ TRP^146^ TYR^37^ VAL^114^ VAL^65^  ASP^64^  SER^66^ THR^12^ ASN^36^ ILE^227^ ALA^183^ SER^39^ HIS^151^ ALA^190^ ALA^193^ GLU^191^ |
| Binding Site2 | GLY156 ARG158 GLY200 THR202 GLU268 GLY197 ARG155 VAL201 ALA198 TRP269 LYS199 ASN271 ALA193 CYS196 ILE270  SER267 | ILE^142^ HIS^248^ SER^250^ ALA^253^ TRP^146^ GLY^252^ VAL^145^ ALA^249^ GLY^251^ SER^141^ SER^143^ PRO^184^ LYS^185^ TYR^196^ ILE^212^ SER^216^ ARG^220^ PRO^221^ GLY^222^  ASP^226^ ILE^227^ VAL^230^ PHE^223^ ALA^183^ PRO^224^ SER^213^ LEU^218^ TYR^154^  THR^186^ MET^188^  TYR^189^ ALA^192^  GLU^95^  ALA^193^ HIS^151^ ASP^209^ ASN^225^ ILE^18^ PRO^217^ GLY^219^ ASP^187^ ALA^190^ |
| Binding Site3 | GLY40 GLY116 MET215 | LYS^79^ ARG^84^ LEU^85^ |
