# Supplementary material for: In silico Prediction, Characterization, Molecular Docking, and Dynamic Studies on Fungal SDRs as Novel Targets for Searching Potential Fungicides Against Fusarium Wilt in Tomato
Source: Front Pharmacol. 2018 Oct 22;9:1038. doi: 10.3389/fphar.2018.01038 (PMC6204350; doi:10.3389/fphar.2018.01038)
Supplement: Supplementary file 5 [file Table_5.DOCX]

| 1. | Oxathiopiprolin | LEU-100, VAL-103, ILE-108, LEU-112, VAL-116, TRP-146, GLY-147, VAL-148, PRO-149, ARG-150, HIS-151, ALA-152, LEU-153, SER-155, ALA-156, SER-157 AND ALA-160 |
| --- | --- | --- |
| 2. | **Famoxadone** | GLY-13, SER-15, ARG-16, GLY-17, ILE-18, GLY-19, TYR-37, VAL-38, ASN-91, SER-92, GLY-93, ILE-94, GLU-95, ILE-139, SER-140, SER-141, ILE-142, SER-143, TYR-154, LYS-158, PRO-184, LYS-185, THR-186, ASP-187, MET-188, TYR-189, ALA-192, ALA-193 AND TYR-196 |
| 3. | Tolprocarb | ILE-108, VAL-111, LEU-112, VAL-116, LEU-153, ALA-156, SER-157, ALA-159, ALA-160, GLY-163 AND MET-164 |
| 4. | Metiram | ILE-18, ASN-91, SER-92, GLY-93, GLU-95, ILE-139, SER-140, SER-141, SER-143, HIS-151, TYR-154, LYS-158, PRO-184, LYS-185, MET-188, TYR-189, ALA-192, TYR-196 |
| 5. | Dithane | ILE-18, ASN-91, SER-92, GLY-93, GLU-95, ILE-139, SER-140, SER-141, SER-143, HIS-151, TYR-154, LYS-158, PRO-184, LYS-185, THR-186, MET-188, TYR-189, ALA-192, TYR-196 |
| 6. | Pyraclostrobin | ILE-18, ASN-91, SER-92, GLY-93, ILE-94, GLU-95, ILE-139, SER-140, SER-141, ILE-142, SER-143, TYR-154, LYS-158, PRO-184, LYS-185, THR-186, MET-188, TYR-189, ALA-192, ALA-193, TYR-196 |
| 7. | Pterostilbin | ILE-18, ASN-91, SER-92, GLY-93, ILE-94, GLU-95, ILE-139, SER-140, SER-141, ILE-142, SER-143, TYR-154, LYS-158, PRO-184, LYS-185, THR-186, MET-188, TYR-189, ALA-192, ALA-193, TYR-196 |
| 8. | Tolclofos-Methyl | ILE-18, ASN-91, GLY-93, GLU-95, ILE-139, SER-140, SER-141, ILE-142, SER-143, TYR-154, LYS-158, ALA-183, PRO-184, LYS-185, THR-186, MET-188, TYR-189, ALA-192, TYR-196 |
| 9. | Fluberidazole | ILE-18, ASN-91, GLY-93, GLU-95, ILE-139, SER-140, SER-141, ILE-142, SER-143, TYR-154, LYS-158, PRO-184, LYS-185, THR-186, MET-188, TYR-189, ALA-192, TYR-196 |
| 10. | Cymoxanil | ILE-18, ASN-91, SER-92, GLY-93, GLU-95, ILE-139, SER-140, SER-141, ILE-142, SER-143, TYR-154, LYS-158, PRO-184, LYS-185, THR-186, MET-188, TYR-189, ALA-192, TYR-196 |
| 11. | Carbendazim | ILE-18, ASN-91, GLY-93, GLU-95, ILE-139, SER-140, SER-141, ILE-142, 143,TYR-154, LYS-158, PRO-184, LYS-185, THR-186, MET-188, TYR-189, ALA-192, TYR-196 |
| 12. | Coumarin | ILE-18, GLU-95, ILE-139, SER-141, ILE-142, SER-143, TYR-154, LYS-158, PRO-184, LYS-185, THR-186, MET-188, TYR-189, ALA-192, TYR-196 |
| 13. | Triazoquinoline | ILE-18, GLU-95, ILE-139, SER-140, SER-141, ILE-142, SER-143, HIS-151, TYR-154, LYS-158, PRO-184, LYS-185, THR-186, MET-188, TYR-189, ALA-192, TYR-196 |
| 14. | Fludioxonil | ILE-18, ASN-91, SER-92, GLY-93, GLU-95, ILE-139, SER-140, SER-141, ILE-142, SER-143, TYR-154, LYS-158, PRO-184, LYS-185, THR-186, MET-188, TYR-189, ALA-192, TYR-196 |
| 15. | Iprodione | GLY-17, ILE-18, GLY-19, ASN-91, SER-92, GLY-93, ILE-94, GLU-95, ILE-139, SER-140, SER-141, ILE-142, SER-143, TYR-154, LYS-158, PRO-184, LYS-185, THR-186, MET-188, TYR-189, ALA-192, TYR-196 |
| 16. | Ethyl Phosphonate | GLU-95, SER-141, SER-143, TYR-154, PRO-184, LYS-185, THR-186, MET-188, TYR-189, ALA-192, TYR-196 |
| 17. | Prochloraz | GLY-13, ARG-16, GLY-17, ILE-18, GLY-19, ASN-91, SER-92, GLY-93, ILE-94, GLU-95, ILE-139, SER-140, SER-141, ILE-142, SER-143, HIE-151, TYR-154, LYS-158, PRO-184, LYS-185, THR-186, ASP-187, MET-188, TYR-189, ALA-192 AND TYR-196 |
| 18. | Prothioconazole | ARG-16, GLY-17, ILE-18, ASN-91, SER-92, GLY-93, GLU-95, ILE-139, SER-140, SER-141, ILE-142, SER-143, HIE-151, TYR-154, LYS-158, ALA-183, PRO-184, LYS-185, THR-186, ASP-187, MET-188, TYR-189, ALA-192 AND TYR-196 |
| 19. | Benomyl | ILE-18, ASN-91, SER-92, GLY-93, GLU-95, ILE-139, SER-140, SER-141 ILE-142, SER-143, TRP-146, TYR-154, LYS-158, PRO-184, LYS-185, THR-186, MET-188, TYR-189, ALA-192, TYR-196 |
